# Phase 1/2 study of INCAGN01876, an anti-glucocorticoid-induced tumor necrosis factor receptor agonist monoclonal antibody, plus immunotherapy for advanced cancers

**DOI:** 10.1093/oncolo/oyag257

**Published:** 2026-07-30

**Authors:** Omid Hamid, Frederic Forget, Melissa Johnson, Thomas J George, Nawel Bourayou, Sonia Ioannidis, Feng Zhou, Hong Yang, Zhiwan Dong, Martin E Gutierrez

**Affiliations:** The Angeles Clinic and Research Institute, A Cedars Sinai Affiliate, Los Angeles, CA, 90025, United States; Centre Hospitalier de l‘Ardenne, Libramont-Chevigny, 6800, Belgium; Sarah Cannon Research Institute, Nashville, TN, 37203, United States; University of Florida, Gainesville, FL, 32610, United States; Incyte Biosciences International Sàrl, Morges, 1110, Switzerland; Incyte Biosciences International Sàrl, Morges, 1110, Switzerland; Incyte Corporation, Wilmington, DE, 19803, United States; Incyte Corporation, Wilmington, DE, 19803, United States; Incyte Corporation, Wilmington, DE, 19803, United States; John Theurer Cancer Center, Hackensack University Medical Center, Hackensack, NJ, 07601, United States

**Keywords:** nivolumab, ipilimumab, monoclonal antibody, head and neck neoplasms, neoplasm metastasis, immunotherapy

## Abstract

**Background:**

Modulating tumor-mediated immunosuppression with immunotherapies is an effective therapeutic approach for solid tumors. INCAGN01876 is a humanized IgG1 anti-glucocorticoid-induced tumor necrosis factor receptor (GITR) monoclonal antibody. This phase 1/2 trial evaluated INCAGN01876 plus nivolumab and/or ipilimumab for advanced malignancies.

**Methods:**

In phase 1 (dose escalation), patients received various INCAGN01876 plus nivolumab and/or ipilimumab regimens. In phase 2 (dose expansion), patients with select tumors received INCAGN01876 plus ipilimumab (treatment group [TG] C2) or INCAGN01876 plus nivolumab (TGF). Primary endpoints: safety (phase 1), objective response rate (ORR; phase 2).

**Results:**

Overall, 145 patients were enrolled: 51 and 94 in phases 1 and 2, respectively (TGC2, *n* = 8; TGF, *n* = 86 [squamous cell carcinoma of the head and neck, SCCHN, *n* = 46]). Four patients had dose-limiting toxicities; maximum tolerated dose was not reached; INCAGN01876 300 mg Q2W was selected as the recommended phase 2 dose based on safety with nivolumab and/or ipilimumab and safety and pharmacokinetic/pharmacodynamic monotherapy data from the INCAGN 1876-101 phase 1 study. INCAGN01876-related treatment-emergent adverse events (TEAEs) occurred in 62.8% of patients (most commonly pruritus, 16.6%); grade ≥3, 13.8%. Immune-related TEAEs occurred in 31.0% of patients (most frequently pruritus, 11.7%) most were (75.6%) grade 1/2 events. Antitumor activity was observed in TGF cohorts with SCCHN or cervical cancer (ORR 23.9% and 16.7%, respectively).

**Conclusion:**

INCAGN01876 plus nivolumab and/or ipilimumab was generally well tolerated, with a safety profile consistent with previous reports. This observation, along with encouraging antitumor activity in SCCHN and cervical cancer, supports development of INCAGN01876 combined with immune checkpoint inhibitors.

Implications for Practice Simultaneous targeting of multiple immune checkpoints is a proposed solution to ICI treatment resistance (eg, PD-1/PD-L1 and CTLA-4). GITR agonists act through promoting effector T-cell activity while inhibiting regulatory T-cell immunosuppressive activity. The distinct mechanisms of action from PD-1 and CTLA-4 may augment their effects, offering a novel treatment option. Here, INCAGN01876, a humanized GITR-targeting agonistic antibody, was well tolerated with nivolumab (anti-PD-1) and/or ipilimumab (anti-CTLA-4). The demonstrated antitumor activity of INCAGN01876 with nivolumab in patients with SCCHN and cervical cancer warrants future studies of INCAGN01876 with ICIs focused on these tumors.

## Introduction

Immune checkpoint receptors are established gatekeepers for immune regulation that can trigger or inhibit immune responses.^1^^,^^2^ Modulation of these checkpoints to diminish inflammatory processes or escalate an immune response is an established approach in cancer immunotherapy.^3^^,^^4^ The development of immune checkpoint inhibitors (ICIs), including anti-programmed cell death protein 1 (PD-1), programmed cell death ligand 1 (PD-L1), and anticytotoxic T-lymphocyte-associated protein 4 (CTLA-4) monoclonal antibodies (mAbs),^1^^,^^2^^,^^5^^,^^6^ has advanced cancer treatment beyond traditional surgery, chemotherapy, and radiotherapy.^5^^,^^7^ However, primary and acquired ICI resistance remains a clinical challenge.^7^ Beyond inhibitory checkpoints, costimulatory receptors represent potential therapeutic targets for overcoming tumor resistance to immune surveillance and ICI resistance.^8^

Tumor necrosis factor super family (TNFSF) member glucocorticoid-induced tumor necrosis factor receptor (GITR)^9^ is constitutively expressed at high levels on CD4^+^CD25^+^FoxP3^+^ regulatory T cells (Tregs). GITR expression is upregulated upon activation of CD4^+^ and CD8^+^ T cells^9–13^ and is observed on T effector (Teff) cells and natural killer cells.^14–17^ GITR functions as a costimulator receptor, enhancing activation and survival of these immune cells.^10–12^^,^^18^^,^^19^ Engagement by its ligand, GITRL,^17^^,^^20^ promotes T-cell proliferation, differentiation, survival, and increased cytokine-mediated inflammatory responses.^15^^,^^18^^,^^21^^,^^22^ GITR has been identified on tumor-infiltrating Tregs, which contribute to suppression of the antitumor response.^23^ However, GITR costimulation during T-cell activation can inhibit this immune suppression, potentially via a transient loss of FoxP3.^24^ GITR can also selectively deplete Tregs within tumors and modulate their function toward a Teff phenotype.^25^ Studies of human tissues, including lung cancers, renal cell carcinoma, head and neck cancer, and melanoma, identified GITR on tumor-infiltrating lymphocytes (TILs).^26^ Notably, GITR expression varies with tumor type, with GITR expression higher on Tregs versus Teff cells in all studied tumors.^26^

Activation of costimulatory pathways through GITR is therefore considered a promising therapeutic target^9^ and may potentiate the efficacy of other anticancer therapies. GITR engagement synergistically prolonged survival when combined with anti-PD-1 mAbs in both cisplatin- and paxlitaxel-treated mice,^9^^,^^27^ and enhanced tumor growth inhibition when combined with both TGFβ inhibitor and gemcitabine in humanized mouse xenografts.^9^^,^^28^ Improved antitumor activity was also observed in mouse models targeted with combined anti-CTLA-4/anti-GITR treatment versus either monotherapy.^9^^,^^29^

INCAGN01876 is a humanized IgG1 anti-GITR mAb^30^^,^^31^ designed to induce antitumor immunity by promoting Teff activity while inhibiting Treg immunosuppressive activity. Preclinical studies showed INCAGN01876 selectively binds to human GITR and functions as a GITR agonist.^30^^,^^31^ A previous phase 1/2 study (INCAGN 1876-101; NCT02697591) showed INCAGN01876 monotherapy was generally well tolerated at doses ≤10 mg/kg in patients with advanced/metastatic solid tumors; however, as seen in other studies with GITR monotherapies,^32–35^ antitumor activity was limited, with only 2/100 evaluable patients achieving a partial response (PR).^36^ Combining ICIs, such as anti-CTLA-4 ipilimumab and anti-PD-1 nivolumab, with INCAGN01876 may enhance antitumor immune responses and provide additional clinical benefit in advanced/metastatic cancers.^9^^,^^37^

We report the results of a phase 1/2 study that assessed safety, tolerability, recommended phase 2 dose (RP2D), and preliminary efficacy of INCAGN01876 in combination with nivolumab and/or ipilimumab in patients with advanced malignancies.

## Methods

### Study design and patients

This global, multicenter, open-label, nonrandomized phase 1/2 dose-escalation/dose-expansion study (ClinicalTrials.gov: NCT03126110; EudraCT: 2016-004989-25) enrolled patients (≥18 y) with locally advanced (not amenable to curative resection) or metastatic disease. Patients in phase 1 had immunogenic tumors known to be effective targets for PD-1/PD-L1 therapy; other tumor types were acceptable with the sponsor’s medical monitor approval. Patients had progressed on or were refractory to prior treatment (unlimited treatment lines allowed) or refused standard treatment. Patients had confirmed measurable disease per Response Evaluation Criteria in Solid Tumors (RECIST) v1.1 and an Eastern Cooperative Oncology Group performance status ≤1. Patients in phase 2 had advanced/metastatic cervical cancer, gastric cancer (including stomach, esophageal, and gastroesophageal junction adenocarcinoma [GEJ]), squamous cell carcinoma of head and neck (SCCHN), or PD-1/PD-L1 relapsed melanoma. Patients with cervical cancer, gastric cancer, and SCCHN had no prior immunotherapy. An additional prespecified “biopsy” cohort was enrolled in phase 2, in which mandatory pretreatment and on-treatment biopsies were collected to evaluate changes in the tumor microenvironment during treatment (Supplementary Methods).

### Ethics approval

The protocol was approved by an Institutional Review Board/Independent Ethics Committee prior to patient enrollment (Supplementary Methods**)**. The study was conducted in accordance with the International Council for Harmonisation Guideline for Good Clinical Practice, the principles of the Declaration of Helsinki, and all local laws and regulations. Patients provided informed consent before screening.

### Treatments

INCAGN01876 was administered intravenously (IV) over a 30-minute period on day 1 of each cycle, followed by a 30-minute wait before nivolumab and/or ipilimumab treatment. Nivolumab and ipilimumab were given IV per the prescribing information.^38^^,^^39^ In phase 1, patients received INCAGN01876 at a range of doses based on safety data from the INCAGN 1876-101 monotherapy study.^36^ Nivolumab and/or ipilimumab were administered either concurrently or sequentially. Doses were escalated using a 3 + 3 + 3 design to determine the INCAGN01876 maximum tolerated dose (MTD) or pharmacologically active dose (PAD) (Supplementary Methods).

**Figure 1.**
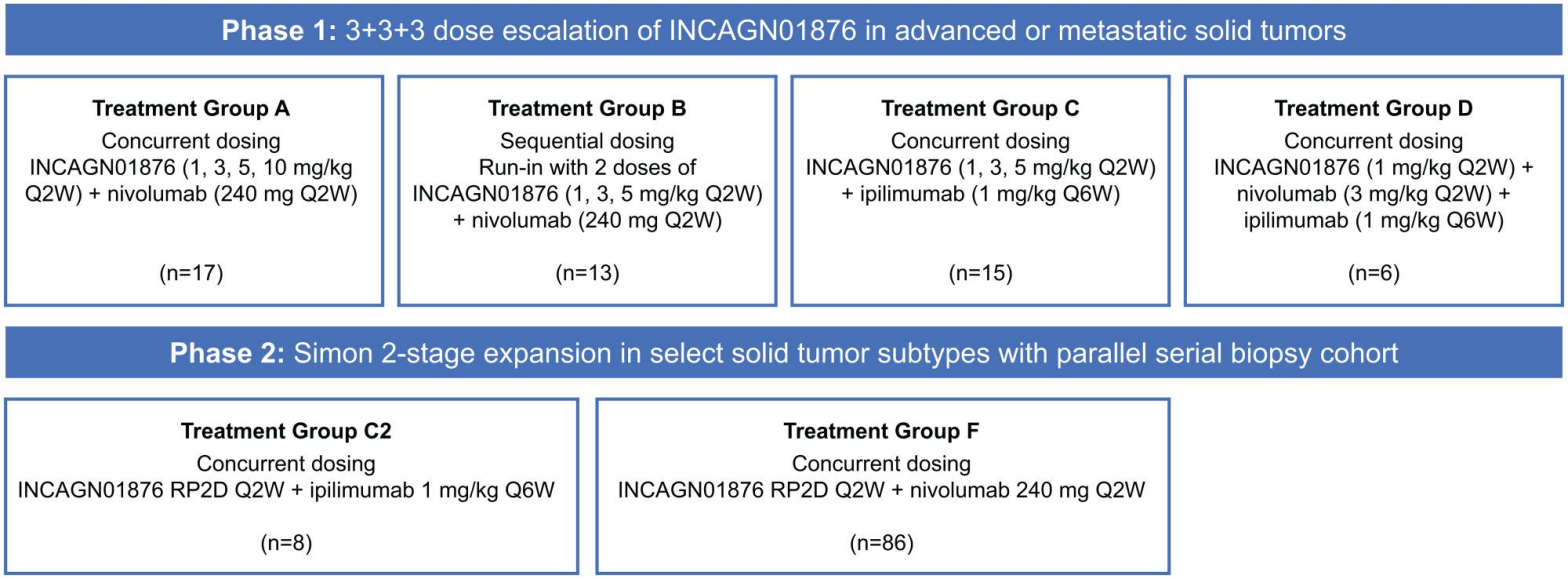
Study design. Abbreviations: Q2W, once every 2 weeks; Q6W, once every 6 weeks; RP2D, recommended phase 2 dose.

Patients were assigned to 4 treatment groups (TGs) (Figure 1): TGA (concurrent dosing)—INCAGN01876 1.0-10.0 mg/kg every 2 weeks (Q2W) plus nivolumab 240 mg Q2W; TGB (sequential dosing)—2 doses of INCAGN01876 1.0-5.0 mg/kg Q2W plus nivolumab 240 mg Q2W from cycle 3 day 1; TGC (concurrent dosing)—INCAGN01876 1.0-5.0 mg/kg Q2W plus ipilimumab 1 mg/kg Q6W; TGD (concurrent dosing)—INCAGN01876 1.0 mg/kg Q2W plus nivolumab 3 mg/kg Q2W and ipilimumab 1 mg/kg Q6W.

Phase 2 commenced following determination of the MTD or PAD of INCAGN01876 in phase 1. Patients with select advanced/metastatic tumor types were assigned to 2 concurrent dosing TGs (Figure 1), and the INCAGN01876 dose was expanded in tumor-specific cohorts: TGC2—patients with PD-1/PD-L1 relapsed melanoma received INCAGN01876 Q2W at the RP2D selected in phase 1 plus ipilimumab 1 mg/kg Q6W; TGF—patients with cervical cancer, gastric cancer, SCCHN, or PD-1/PD-L1 relapsed melanoma received INCAGN01876 Q2W at the RP2D plus nivolumab 240 mg Q2W. Per the Simon 2-stage design,^40^ tumor-specific cohorts with sufficient responses observed at the end of stage 1 continued into stage 2 (Supplementary Methods). An integrated Bayesian analysis of tumor and dosing strategies was also used to determine responses/treatment activity for cervical cancer, gastric cancer, and SCCHN cohorts based on Simon et al., 2016.^41^

### Objectives and assessments

The primary objectives of phase 1 were to evaluate the safety, tolerability, and dose-limiting toxicities (DLTs), and define the RP2D of INCAGN01876 administered in combination with immunotherapies. The primary objective of phase 2 was evaluation of INCAGN01876 efficacy in combination with immunotherapies by assessing overall response rate (ORR) per RECIST v1.1, defined as the percentage of patients with a complete response (CR) or PR. Secondary objectives in phases 1 and 2 were assessment of ORR, duration of response (DOR), disease control rate (DCR), and progression-free survival (PFS) per RECIST v1.1 or modified RECIST v1.1, and 1- and 2-year overall survival (OS). An additional secondary objective in phase 2 was evaluation of INCAGN01876 safety and tolerability in combination with immunotherapy. Exploratory endpoints in phases 1 and 2 were INCAGN01876 pharmacokinetics (PK), including maximum observed serum concentration (C_max_), time to maximum serum concentration (t_max_), minimum observed serum concentration over the dose interval (C_min_)_,_ area under the serum concentration-time curve from time = 0 to the last measurable concentration at time = t (AUC_0-t_), immunogenicity (defined as occurrence of antidrug antibodies [ADA] to INCAGN01876 when given in combination with immune therapies), and peripheral blood and tissue biomarkers when given in combination therapy.

Safety and tolerability were assessed from treatment-emergent adverse events (TEAEs), laboratory values, vital signs, and 12-lead ECGs. TEAE severity and assessment of DLTs by investigators (Table S1) were based on Common Terminology Criteria for Adverse Events v4.03. ORR was measured by assessment of tumor response by radiological imaging. Baseline scans were performed using computed tomography or magnetic resonance imaging; other methods were allowed in cases of contrast allergy or with the sponsor’s medical monitor approval (Supplementary Methods).

### Plasma protein biomarker analysis and whole-blood T-cell population profiling

A multiplex Proximity Extension Assay (Olink Proteomics, Watertown, MA, USA) was used to determine immune and nonimmune plasma proteins. Biomarkers were identified by a matched pair of antibodies coupled to unique partially complementary oligonucleotides and quantified by quantitative real-time polymerase chain reaction. Treatment effects on biomarkers were assessed by comparing baseline (cycle 1 day 1) values with on-treatment values at cycle 1 day 2, cycle 1 day 8, and/or cycle 2 day 1.

Whole-blood and plasma samples were collected and analyzed centrally for immune cell profiling; whole-blood samples were collected for correlative DNA and RNA analysis (Supplementary Methods). Cellular biomarkers in peripheral blood were analyzed using multicolor flow cytometry (FC). Frequencies of immune cells were measured at screening and at various time points on treatment. T-cell and Treg proliferation was analyzed using FC panels (CellCarta, IL, USA).

### Immunohistochemistry analysis of GITR and PD-L1 expression in pretreatment tumor biopsy samples

Tumor biopsy samples were collected at screening (baseline) and on treatment for patients with SCCHN during phase 2 (Supplementary Methods). Potential correlations between PD-L1 tumor proportion score (TPS) and GITR TPS were analyzed by immunohistochemistry. The impact of GITR TPS on primary and secondary endpoints was analyzed ad hoc.

### Statistical analyses

In phase 1, the sample size was the total number of patients enrolled in dose levels sufficient to evaluate the MTD and/or PAD. In phase 2, the sample size was guided by the Simon 2-stage design for each tumor-specific cohort. Patient characteristics, safety, and study drug administration data were analyzed using the full analysis set (FAS, all patients enrolled who received ≥1 dose of INCAGN01876, nivolumab, or ipilimumab). TEAEs were summarized with descriptive statistics using Medical Dictionary for Regulatory Activities (MedDRA) version 24.0. Efficacy was evaluated in the response-evaluable population (all patients enrolled who received ≥1 dose of INCAGN01876, nivolumab, or ipilimumab); completed a baseline scan; and met ≥1 of the following criteria: ≥1 postbaseline scan, enrolled in the study for ≥64 days of follow-up, or discontinued treatment. PFS, DOR, OS, and duration of disease control distributions were estimated using the Kaplan-Meier method. The PK/ADA-evaluable population included patients who received ≥1 INCAGN01876 dose and had ≥1 postdose PK/ADA sample collected and analyzed. Determination of significance of the effect of combining INCAGN01876 and nivolumab on T-cell proliferation was by paired *t* test. PK parameters were summarized based on patient samples collected immediately postinfusion during cycles 1 and 6 (Supplementary Methods**)**.

## Results

### Patients

A total of 145 patients were enrolled at 32 centers (Australia, *n* = 13; Belgium, *n* = 27; Spain, *n* = 21; USA, *n* = 84) from April 25, 2017, to May 20, 2022. Fifty-one patients were enrolled in phase 1 (TGA, *n* = 17; TGB, *n* = 13; TGC, *n* = 15; TGD, *n* = 6), and 94 were enrolled in phase 2 (TGC2, *n* = 8; TGF, *n* = 86). The SCCHN cohort (TGF) met the prerequisite number of responses at the end of Simon stage 1 (*n* = 10) (Supplementary Methods); enrollment was completed for a total of 46 patients. Enrollment of other tumor-specific cohorts was terminated early. Therefore, although data from all study cohorts are presented, data from patients with SCCHN in TGF are presented in further detail.

All 145 patients enrolled were included in the FAS and response-evaluable population. In phase 1, median age ranged from 59.0 to 66.0 years across TGA-TGD (Table 1). In phase 2, median age was 70.5 years and 57.0 years in TGC2 and TGF, respectively. Nearly all patients enrolled had received prior cancer therapy (140/145 [96.6%]), most commonly 1 prior line (68/145, 46.9%) (Table 1, Supplementary Results). Most patients in phase 1 were PD-1/PD-L1 and CTLA-4 targeted treatment naive (39/51, 76.5%, and 50/51, 91.0%, respectively). In phase 2, 1 patient with SCCHN in TGF had a protocol deviation (prior PD-1/PD-L1 treatment) and was excluded from ad hoc analysis; all other patients in TGF (except with PD-1/PD-L1 relapsed melanoma) were immunotherapy naive. All patients in TGC2 had received prior PD-1/PD-L1 treatment; most had received prior CTLA-4 treatment (5/8, 62.5%). INCAGN01876 exposure for each TG and for TGF cohorts is summarized in Supplementary Results and Table S2.

**Table 1:** Patient demographics and baseline characteristics.

Variable	Phase 1				Phase 2		Total *N* = 145
	Treatment group A	Treatment group B	Treatment group C	Treatment group D	Treatment group F	Treatment group C2	
	(*n* = 17)	(*n* = 13)	(*n* = 15)	(*n* = 6)	(*n* = 86)	(*n* = 8)	
**Age, years, median (range)**	59.0 (22-70)	64.0 (40-82)	66.0 (44-73)	58.0 (42-74)	57.0 (30-77)	70.5 (48-81)	60 (22-82)
**Male, *n* (%)**	5 (29.4)	4 (30.8)	6 (40)	4 (66.7)	53 (61.6)	5 (62.5)	77 (53.1)
**Race, *n* (%)**							
** White**	12 (70.6)	9 (69.2)	12 (80.0)	6 (100.0)	81 (94.2)	8 (100.0)	128 (88.3)
** Black**	3 (17.6)	1 (7.7)	0	0	3 (3.5)	0	7 (4.8)
** Asian**	0	2 (15.4)	2 (13.3)	0	1 (1.2)	0	5 (3.4)
** Other**	2 (11.8)	1 (7.7)	1 (6.7)	0	1 (1.2)	0	5 (3.4)
**ECOG performance status, *n* (%)^a^**							
** 0**	13 (76.5)	5 (38.5)	11 (73.3)	1 (16.7)	35 (40.7)	2 (25.0)	67 (46.2)
** 1**	4 (23.5)	8 (61.5)	3 (20.0)	5 (83.3)	51 (59.3)	6 (75.0)	77 (53.1)
**Prior lines of cancer therapy, *n* (%)**	16 (94.1)	12 (92.3)	15 (100.0)	6 (100.0)	83 (96.5)	8 (100.0)	140 (96.6)
** 0**	1 (5.9)	1 (7.7)	0	0	3 (3.5)	0	5 (3.5)
** 1**	4 (23.5)	3 (23.1)	3 (20.0)	0	52 (60.5)	6 (75.0)	68 (46.9)
** 2**	4 (23.5)	3 (23.1)	2 (13.3)	3 (50.0)	22 (25.6)	2 (25.0)	36 (24.8)
** ≥3**	8 (47.1)	6 (46.2)	10 (66.7)	3 (50.0)	9 (10.5)	0	36 (24.8)
**Prior PD-1/PD-L1 therapy, *n* (%)**	3 (17.6)	2 (15.4)	6 (40.0)	1 (16.7)	5 (5.8)	8 (100.0)	25 (17.2)
**Prior anti-CTLA-4 therapy, *n* (%)**	0	0	1 (6.7)	0	3 (3.5)	5 (62.5)	9 (6.2)

a One patient in treatment group C had an ECOG performance status of 2; the patient had a screening score of 1.

Abbreviations: CTLA-4, cytotoxic T-lymphocyte-associated protein 4; ECOG, Eastern Cooperative Oncology Group; PD-1, programmed cell death protein 1; PD-L1, programmed cell death ligand 1.

### Efficacy

**Figure 2.**
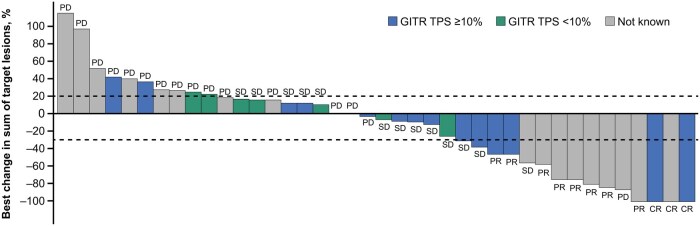
Best percent change from baseline in the sum of target lesions stratified by GITR TPS among patients in treatment group F with SCCHN based on confirmed responses. Patients with GITR TPS ≥10% were considered GITR-positive. Abbreviations: CR, complete response; PD, progressive disease; PR, partial response; SCCHN, squamous cell carcinoma of the head and neck; SD, stable disease; TPS, tumor proportion score.

In phase 2, among patients in TGF with SCCHN (*n* = 46), the ORR was 23.9% (*n* = 11) based on confirmed tumor responses, with the best overall response (BOR) of CR in 3 patients (6.5%) and PR in 8 patients (17.4%) (Table 2). DCR was 54.3%, with a median (95% CI) duration of 253.0 (156.0-not established [NE]) days, DOR was NE (118.0-NE) days, PFS was 115.0 (59.0-174.0) days, and OS was 491.0 (344.0-NE) days. Figure S1 (see online supplementary material for a color version of this figure) shows duration of unconfirmed responses; Figure 2 shows best percent change from baseline in the sum of target lesions based on confirmed responses, stratified by patient GITR TPS status; Figure S2 (see online supplementary material for a color version of this figure) shows percent change from baseline in the sum of target lesions over time. Most patients with confirmed tumor response had 1 or 2 lines of prior therapy. Among 18 patients with cervical cancer in TGF (*n* = 18), the ORR was 16.7%, including a CR in 1 patient (5.6%) and a PR in 2 patients (11.1%). There were no responders in TGC2 (INCAGN01876 plus ipilimumab) or among patients in TGF (INCAGN01876 plus nivolumab) cohorts with gastric cancer, PD-1/PD-L1 relapsed melanoma, or biopsy cohorts. Phase 1 and 2 efficacy endpoint outcomes are summarized in Tables S3 and S4.

**Table 2:** Summary of BOR based on confirmed tumor responses in patients from treatment group F with SCCHN according to RECIST v1.1.

Variable, *n* (%)	Treatment group F (SCCHN cohort) (*n* = 46)	GITR TPS-positive (*n* = 17)	GITR TPS-negative (*n* = 10)	GITR TPS total^a^ (*n* = 30)
**BOR^b^**				
** CR**	3 (6.5)	2 (11.8)	0	2 (6.7)
** PR**	8 (17.4)	2 (11.8)	0	2 (6.7)
** SD**	14 (30.4)	7 (41.2)	5 (50.0)	13 (43.3)
** PD**	16 (34.8)	4 (23.5)	3 (30.0)	9 (30.0)
** NE**	5 (10.9)	2 (11.8)	2 (20.0)	4 (13.3)
**ORR^c^**	11 (23.9)	4 (23.5)	0	4 (13.3)

a Includes 1 patient with a baseline positive GITR score who received prior treatment with pembrolizumab and 2 patients whose GITR tumor score at baseline was NE.

b The BOR was defined as the best confirmed response recorded before and including the first event of PD in the order of CR, PR, SD (≥49 days), PD, and NE.

c A patient was considered as an objective responder if the patient had a best overall response of CR or PR.

Abbreviations: BOR, best overall response; CR, complete response; GITR, glucocorticoid-induced tumor necrosis factor receptor; NE, not evaluable; ORR, objective response rate; PD, progressive disease; PR, partial response; RECIST, Response Evaluation Criteria in Solid Tumors; SCCHN, squamous cell carcinoma of the head and neck; SD, stable disease; TPS, tumor proportion score.

### Safety

TEAEs are summarized in Table 3, Supplementary Results, Tables S5 and S6. DLTs were reported for 1 patient in phase 1 (2.0%, TGD; nonserious Grade 3 pruritus and nonserious Grade 3 rash) and 3 patients in phase 2 (1 patient [12.5%] in TGC2; nonserious Grade 2 penile pain and nonserious Grade 2/3 pruritus, and 2 patients [2.3%] in TGF; nonserious Grade 2/3 rash, serious Grade 3 bronchial obstruction, *n* = 1 patient each). No dose-dependent toxicities were observed, and MTD was not reached. INCAGN01876-related AEs occurred in 62.8% of patients, most commonly pruritus (16.6%), rash (12.4%), and fatigue (12.4%); 13.8% had grade ≥3 INCAGN01876-related TEAEs, with only asthenia, pruritus, and rash occurring in >1 patient. TEAEs led to INCAGN01876 dose interruption, dose delay, and discontinuation in 29.0%, 26.9%, and 9.7% of patients, respectively. Immune-related TEAEs (irTEAEs) occurred in 31.0% of patients, most frequently pruritus (11.7%) and rash (8.3%); most (75.6%) were grade 1/2 events; 12.4% of patients had fatal TEAEs. Any-grade and grade ≥3 TEAEs across all TGs are summarized in Tables S5 and S6; irTEAEs are summarized in Table S7.

**Table 3:** Summary of TEAEs.

Patients with TEAEs, *n* (%)	Phase 1				Phase 2		Total *N* = 145
	Treatment group A (*n* = 17)	Treatment group B (*n* = 13)	Treatment group C (*n* = 15)	Treatment group D (*n* = 6)	Treatment group F (*n* = 86)	Treatment group C2 (*n* = 8)	
**TEAE**	17 (100.0)	13 (100.0)	14 (93.3)	6 (100.0)	85 (98.8)	8 (100.0)	143 (98.6)
** Grade ≥3**	10 (58.8)	5 (38.5)	7 (46.7)	4 (66.7)	55 (64.0)	3 (37.5)	84 (57.9)
**Serious TEAE**	9 (52.9)	5 (38.5)	6 (40.0)	2 (33.3)	43 (50.0)	3 (37.5)	68 (46.9)
**Investigator-assessed INCAGN01876-related TEAE**	8 (47.1)	6 (46.2)	10 (66.7)	5 (83.3)	56 (65.1)	6 (75.0)	91 (62.8)
** Grade ≥3**	0	0	1 (16.7)	2 (33.3)	16 (18.6)	1 (12.5)	20 (13.8)
**Fatal TEAE**	3 (17.6)	1 (7.7)	1 (6.7)	1 (16.7)	11 (12.8)	1 (12.5)	18 (12.4)
**INCAGN01876 dose interruption due to a TEAE**	0	0	4 (26.7)	2 (33.3)	35 (40.7)	1 (12.5)	42 (28.9)
**INCAGN01876 dose delay due to a TEAE**	0	0	3 (20.0)	1 (16.7)	34 (39.5)	1 (12.5)	39 (26.9)
**TEAE leading to discontinuation**	1 (5.9)	0	0	0	12 (14.0)	1 (12.5)	14 (9.7)
**Investigator-assessed immune-related TEAE**	5 (29.4)	5 (38.5)	6 (40.0)	2 (33.3)	24 (27.9)	3 (37.5)	45 (31.0)
** Grade ≥3**	0	0	1 (6.7)	1 (16.7)	8 (9.3)	1 (12.5)	11 (7.6)
**DLT**	0	0	0	1 (16.7)	2 (2.3)	1 (12.5)	4 (2.8)
**Infusion-related reaction**	1 (5.9)	0	1 (6.7)	1 (16.7)	3 (3.5)	0	6 (4.1)

Abbreviations: DLT, dose-limiting toxicity; TEAE, treatment-emergent adverse event.

Among patients in TGF with SCCHN, the most frequently reported any-grade TEAE was fatigue (*n* = 15, 32.6%), and the most common grade ≥3 TEAEs were anemia and dysphagia (*n* = 3, 6.5%; both grade 3). INCAGN01876-related TEAEs were reported by 32 (69.6%) patients, most commonly fatigue (*n* = 8, 17.4%). irAEs were observed in 14 patients (30.4%), most frequently hypothyroidism, decreased lymphocyte count, and pruritus (*n* = 3 each, 6.5%) (Table 4).

**Table 4:** Any-grade TEAEs in ≥5 (10%), grade ≥3 TEAEs and INCAGN01876-related TEAEs occurring in >2 (5%), and all irAEs occurring in patients in treatment group F with SCCHN.

MedDRA preferred term, *n* (%)	TEAEs (*n* = 46)
**Any TEAE**	45 (97.8)
** Fatigue**	15 (32.6)
** Pruritus**	9 (19.6)
** Constipation**	8 (17.4)
** Diarrhea**	8 (17.4)
** Anemia**	7 (15.2)
** Hypothyroidism**	7 (15.2)
** Nausea**	7 (15.2)
** Asthenia**	7 (15.2)
** Pyrexia**	7 (15.2)
** Arthralgia**	7 (15.2)
** Upper respiratory tract infection**	6 (13.0)
** Productive cough**	6 (13.0)
** Rash**	6 (13.0)
** Dysphagia**	5 (10.9)
** Vomiting**	5 (10.9)
** Headache**	5 (10.9)
** Dyspnea**	5 (10.9)
**Grade ≥3 TEAEs^a^**	
** Any grade ≥3 TEAE**	23 (50.0)
** Anemia**	3 (6.5)
** Dysphagia**	3 (6.5)
**INCAGN01876-related TEAEs**	
** Any-grade INCAGN01876-related TEAE**	32 (69.6)
** Fatigue**	8 (17.4)
** Hypothyroidism**	5 (10.9)
** Pruritus**	5 (10.9)
** Rash**	5 (10.9)
** Diarrhea**	4 (8.7)
** Asthenia**	4 (8.7)
** Anemia**	3 (6.5)
** Nausea**	3 (6.5)
** Decreased lymphocyte count**	3 (6.5)
**irAEs**	
** Any-grade irAE**	
** Hypothyroidism**	3 (6.5)
** Decreased lymphocyte count**	3 (6.5)
** Pruritus**	3 (6.5)
** Rash**	2 (4.3)
** Colitis**	1 (2.2)
** Diarrhea**	1 (2.2)
** Pancreatitis**	1 (2.2)
** Pyrexia**	1 (2.2)
** Encephalopathy**	1 (2.2)
** Peripheral motor neuropathy**	1 (2.2)

a All grade ≥3 TEAEs occurring in ≥2 patients were severity grade 3.

Abbreviations: irAE, immune-related adverse event; MedDRA, Medical Dictionary for Regulatory Activities; SCCHN, squamous cell carcinoma of the head and neck; TEAE, treatment-emergent adverse event.

### Analysis of peripheral blood plasma markers and whole-blood immune cell profiling

Evaluation of patients with SCCHN or cervical cancer showed a trend toward upregulation of the interferon (IFN)-γ-inducible chemokines CXCL-9 and CXCL-10, as well as the Teff chemokine CCL17, at both time points analyzed (cycle 1 day 8 and cycle 2 day 1) (Figure S3, see online supplementary material for a color version of this figure).

Analysis of peripheral blood samples from patients with SCCHN showed a significant increase from baseline in T-cell proliferation (CD3^+^ Ki67^+^) at cycle 1 day 8 of INCAGN01876 treatment (first on-treatment time point with sample collection); levels remained significantly elevated up to cycle 8 day 1 (Figure S4, see online supplementary material for a color version of this figure). Significant elevations of frequencies of Tregs in CD3^+^ T cells in peripheral blood were also observed in the first 2 weeks (cycle 1 day 8 and cycle 2 day 1). No elevation in Treg levels was seen from cycle 3 day 1 onward (Figure S5, see online supplementary material for a color version of this figure).

### Immunohistochemistry analysis of GITR and PD-L1 in pretreatment tumor biopsies

**Figure 3.**
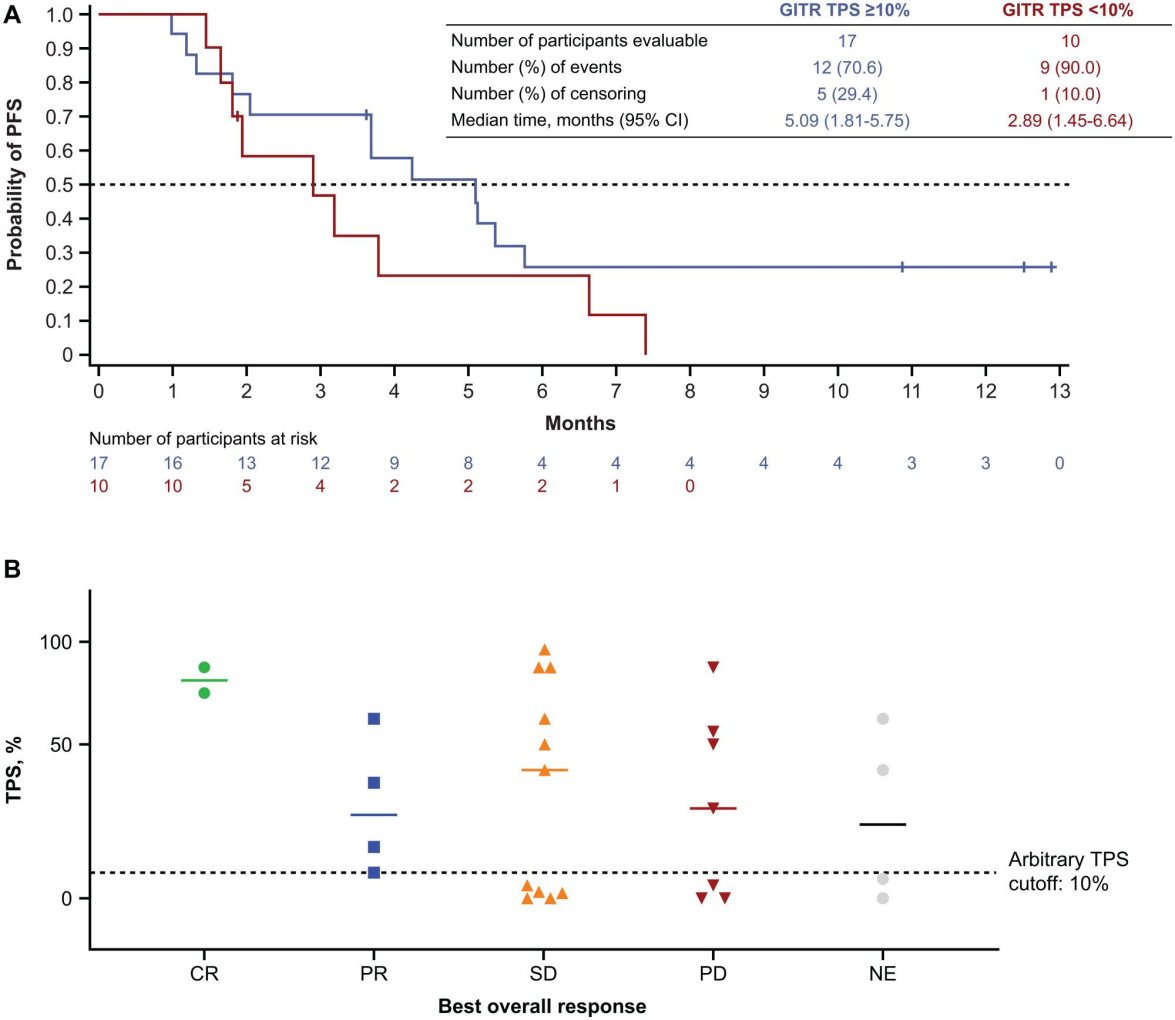
PFS (A) and best overall response (B) stratified by GITR TPS among patients in treatment group F with SCCHN. Patients with GITR TPS ≥10% were considered GITR-positive. Abbreviations: CR, complete response; GITR, glucocorticoid-induced tumor necrosis factor receptor; NE, not evaluable; PD, progressive disease; PFS, progression-free survival; PR, partial response; SCCHN, squamous cell carcinoma of the head and neck; SD, stable disease; TPS, tumor proportion score.

Analysis of pretreatment biopsy samples from PD-1-naive patients with SCCHN receiving INCAGN01876 plus nivolumab showed no correlation between baseline GITR expression (GITR TPS) and PD-L1 expression (linear regression: *P *= .62). A bimodal distribution of GITR TPS values was observed across the samples tested. Consequently, a GITR TPS of 10% was arbitrarily selected as the cutoff to separate the low GITR TPS population from the rest of the patients to assess potential correlations of GITR TPS with response and PFS. Of 17 patients with positive GITR TPS, ORR based on confirmed tumor responses was 23.5%; 2 patients (11.8%) had a BOR of CR, and 2 patients (11.8%) had a BOR of PR (Table 2). Median (range) PFS among patients with <10% and ≥10% GITR TPS was 2.9 months (1.5-6.6) versus 5.1 months (1.8-5.8) (Figure 3A). All GITR-evaluable patients with a clinical response had a GITR TPS of ≥10%. Baseline GITR immune score, GITR total score, PD-L1 TPS, and combined positive scores did not show bimodal distributions, and cutoffs for BOR were therefore based on median values. There was no significant correlation between median score and patient response status (Figure 3B).

### INCAGN01876 pharmacokinetics

INCAGN01876 PK was generally comparable across TGs at the same dose, and for INCAGN01876 monotherapy^36^ compared with INCAGN01876 in combination with immunotherapies (Supplementary Results, Table S8, Figures S6 and S7 [see online supplementary material for a color version of these figures]).

### Antidrug antibodies

Of 145 ADA-evaluable patients, 25 (17.2%) tested positive for ADA across dose levels (all treatment-emergent; none were treatment-boosted). ADA incidence was high at 1 mg/kg (42.1% of patients) and decreased gradually with increasing dose (Supplementary Results, Table S9). The selected RP2D from phase 1 (300 mg Q2W) was associated with a low and acceptable incidence of ADAs (*n* = 10, 10.6%). Similar to previous observations with INCAGN01876 monotherapy,^36^ exposure was substantially reduced in ADA-positive patients (results not shown).

## Discussion

In this phase 1/2 study, INCAGN01876 was generally well tolerated in patients with advanced/metastatic solid tumors when coadministered with nivolumab and/or ipilimumab at the highest doses tested (10 mg/kg Q2W and 5 mg/kg Q2W, respectively). No dose-dependent toxicities were observed, and no new safety findings for INCAGN01876 combination therapy were seen compared with monotherapy in the previous INCAGN 1876-101 study^36^; however, small sample sizes in the dose groups and the single-arm study design limit the ability to test the added benefit of INCAGN01876 in combination therapy.

The RP2D of INCAGN01876 300 mg Q2W in combination with nivolumab and/or ipilimumab was selected based on safety data from the phase 1 dose-escalation portion of this study (*n* = 51), together with safety, PK, and PD data from the previous INCAGN01876 monotherapy study (*N* = 100).^36^ INCAGN01876 PK was dose-proportional across TGs; PK profiles for combination regimens were generally comparable with monotherapy,^36^ suggesting INCAGN01876 PK is not impacted by nivolumab or ipilimumab. The RP2D was associated with a reduced incidence of ADAs compared with lower doses (eg, the incidence of ADAs with 5 mg/kg INCAGN01876, which would correlate to 350 mg for a 70-kg person, was lower than with 1 mg/kg), which is a potential benefit given ADAs were generally associated with a significant reduction in INCAGN01876 exposure.^36^ The reason for the dose-related difference in ADA is unclear, and interpretation is challenged by the small sample size at 5 mg/kg. Use of a fixed dosage also provides several advantages versus weight-based dosing, including convenience of preparation and administration, reduced errors in preparation calculations, and minimization.

INCAGN01876 demonstrated promising antitumor activity in combination with nivolumab in PD-1-naïve patients with SCCHN (ORR 23.9%); responses were considered durable as the median DOR was not reached. INCAGN01876 in combination with nivolumab was associated with more limited antitumor activity in patients with cervical cancer (ORR 16.7%). In cervical cancer, antitumor activity associated with ICI monotherapy has also been reported to be modest, with single-agent activity recorded at levels consistent with that observed here; ORR: 12%-17% for pembrolizumab^42^^,^^43^; 4% for nivolumab^44^; 2.9% for ipilimumab.^45^ No antitumor activity were observed in patients with other tumor types receiving INCAGN01876 plus nivolumab, or in patients receiving INCAGN01876 plus ipilimumab. Median PFS with INCAGN01876 and nivolumab was longer in patients with SCCHN (115 days; 3.8 months) and cervical cancer (102 days; 3.4 months) versus other tumor types (range, 55-87 days; 1.8-2.9 months). Whereas differences in study design and patient populations render cross-trial comparisons difficult, the median PFS observed with INCAGN01876 and nivolumab in patients with SCCHN is consistent with those reported previously for single-agent nivolumab (median PFS, 2.1 months)^46^ and pembrolizumab (median PFS, 2.0 months).^47^ The previously reported PFS associated with ICI monotherapy in patients with cervical cancer is more modest, with single-agent activity recorded at levels consistent with that observed for INCAGN01876 in the current study (pembrolizumab, median PFS, 2.0-2.1 months^42^^,^^43^; nivolumab, 3.5 months^44^; ipilimumab, 2.5 months).^45^ The lack of an INCAGN01876 monotherapy arm in the current study limits comparisons with these studies. A controlled trial is warranted to clarify the PFS benefit of adding INCAGN01876 to nivolumab in SCCHN.

The mechanism underlying the improved efficacy in SCCHN versus other tumors is unclear and warrants further investigation. One explanation might be differences in GITR or FoxP3 expression levels across tumor types.^26^ Although GITR expression has been investigated in a range of tumor types,^26^^,^^48^ there is scant literature comparing GITR levels in SCCHN and cervical cancer with other tumor types. In a study of tissue samples from 76 patients with NSCLC, CRC, bladder cancer, or SCCHN, GITR, and FoxP3 levels were correlated in most patients.^49^ GITR levels were similar across tumors, whereas FoxP3 levels were significantly higher in SCCHN versus NSCLC and bladder tumors.^49^ Thus, increased GITR activity in SCCHN may be due to Tregs having a more dominant role in SCCHN versus other tumors. In human tissues, GITR expression on TILs has only been reported for limited tumor types^26^^,^^33^^,^^48^ and remains poorly characterized.^26^ The present study did not enroll a control arm of patients with PD-1-refractory SCCHN (although all responders were PD-1 naïve); therefore, it is unclear if PD-1 responsiveness is required for combination treatment efficacy.

Notably, an ad hoc analysis of the 17 immunotherapy-naïve patients showed that all responders had a GITR TPS ≥10%, suggesting that GITR TPS could be a potential biomarker for identifying patients who might derive benefit from INCAGN01876 plus nivolumab. Because several nonresponders with progressive disease and stable disease also had high GITR TPS, a suitably powered prospective biomarker trial to confirm an efficacy-GITR TPS correlation may be warranted. Median PFS was also higher in patients with GITR TPS ≥10% versus <10%, although the difference was not significant, again warranting a larger controlled and suitably powered biomarker trial.

Differentiating the immunogenic effects of INCAGN01876 on patient immune cells from those of nivolumab is precluded by single-arm study design. Nivolumab has been shown to increase the proportion of CD4^+^ cells expressing FoxP3 as well as the proportion of CD8^+^ cells.^50^ Notably, no increase was observed in the previous INCAGN01876 monotherapy study^36^ suggesting that the effects on T-cell proliferation observed here may be primarily driven by nivolumab. CD8^+^ T-cell proliferation in peripheral blood has also been reported with anti-PD-1 therapies, including nivolumab and pembrolizumab.^51^ The depletion of Tregs by INCAGN01876 in this study is consistent with that previously observed with INCAGN01876 monotherapy^36^; for which significant, albeit transient, Treg depletion was seen at an early time point (cycle 1 day 7). INCAGN01876 monotherapy was also associated with T-cell activation, a trend of a simultaneous decrease in GITR^+^ Treg expression, and an increase in CD8^+^ Ki67^+^ T cells.^36^ Notably, previous studies report increases in Tregs with nivolumab,^50^ which may have, at least in part, abrogated early INCAGN01876-induced decreases in Tregs when administered in combination. The observed increases in CXCL-9 and CXCL-10 levels with INCAGN01876 in combination with nivolumab are also consistent with the results of previous studies.^52^ Of note, no significant increases in CXCL-9 and CXCL-10 levels were observed previously with INCAGN01786 monotherapy^36^ again suggesting the increases seen here may be primarily driven by nivolumab. Furthermore, treatment with INCAGN01786 monotherapy^36^ significantly increased levels of CCL17, a chemokine for effector T cells and Tregs expressing CCR4,^53^ whereas this was not observed for the anti-PD-1 antibody retifanlimab (Incyte Corporation, data on file). Together, these studies suggest CCL17 is potentially a GITR-specific marker; however, the question whether GITR expression correlates with clinical efficacy requires further investigation. No correlation between GITR expression (GITR TPS) and PD-L1 expression was observed here.

In conclusion, INCAGN01876 300 mg Q2W demonstrated promising antitumor activity with nivolumab 240 mg Q2W, particularly in PD-1-naïve SCCHN; no new safety findings with INCAGN01876 combination therapy were reported. Prospective randomized controlled studies are warranted to determine the benefit of combining INCAGN01876 with nivolumab for SCCHN, particularly in patients with high GITR expression on their tumor cells.

## Supplementary Material

oyag257_Supplementary_Data

## Data Availability

Incyte Corporation (Wilmington, DE, USA) is committed to data sharing that advances science and medicine while protecting patient privacy. Qualified external scientific researchers may request anonymized datasets owned by Incyte for the purpose of conducting legitimate scientific research. Researchers may request anonymized datasets from any interventional study (except phase I studies) for which the product and indication have been approved on or after January 1, 2020, in at least 1 major market (eg, US, EU, JPN). Data will be available for request after the primary publication or 2 years after the study has ended. Information on Incyte’s clinical trial data sharing policy and instructions for submitting clinical trial data requests is available at: https://www.incyte.com/Portals/0/Assets/Compliance%20and%20Transparency/clinical-trial-data-sharing.pdf?ver=2020-05-21-132838-960.
