# Supplementary material for: Phase 1/2 study of INCAGN01876, an anti-glucocorticoid-induced tumor necrosis factor receptor agonist monoclonal antibody, plus immunotherapy for advanced cancers
Source: Oncologist. 2026 Jul 30;31(9):oyag257. doi: 10.1093/oncolo/oyag257 (PMC13451089; doi:10.1093/oncolo/oyag257)

Supplementary Material

Phase 1/2 study of INCAGN01876, an anti-GITR agonist monoclonal antibody, plus immunotherapy in patients with advanced cancers

Omid Hamid[1], Frederic Forget[2], Melissa Johnson[3], Thomas J. George[4], Nawel Bourayou[5], Sonia Ioannidis[5], Feng Zhou[6], Hong Yang[6], Zhiwan Dong[6], Martin E. Gutierrez[7,*]

Contents

Supplementary Methods **1**

Patients **1**

Additional exclusion criteria 1

Ethics 1

Dose-escalation rules 2

Simon 2-stage design 2

Integrated Bayesian analysis 3

Biopsy cohorts 3

Tumor imaging 3

Pharmacokinetic (PK) and antidrug antibody (ADA) analyses 4

Plasma protein biomarker analysis and whole-blood T-cell population profiling 4

Immunohistochemistry analysis of glucocorticoid-induced tumor necrosis factor receptor (GITR) and PD-L1 in pretreatment tumor biopsy samples 4

Statistical analyses 4

Supplementary Results **6**

Patients 6

Exposure 6

Safety 6

PK analyses 6

ADA analyses 7

Supplemental Tables **8**

Table S1. Definition of DLTs 8

Table S2. Summary of exposure. 9

Table S3. Summary of efficacy endpoints. Data represent unconfirmed tumor responses (phase 1) and confirmed tumor responses (phase 2) according to RECIST v1.1. 10

Table S4. Summary of efficacy endpoints in the phase 2 treatment group F cohorts other than SCCHN. 11

Table S5. TEAEs. 12

Table S6. Grade ≥3 TEAEs. 14

Table S7. irAEs.[a] 15

Table S8. INCAGN01876 PK.[a] 16

Table S9. Summary of immunogenicity results in INCAGN01876. 17

Supplemental Figures **18**

Figure S1. Duration of unconfirmed response by RECIST v1.1 (phase 2) among patients in treatment group F with SCCHN. 18

Figure S2. Spider plot of percentage change from baseline in the sum of target lesions over time among patients in treatment group F with SCCHN. 19

Figure S3. Effect of treatment with INCAGN01876 with nivolumab on cytokine expression among patients in treatment group F with SCCHN or cervical cancer. 20

Figure S4. Effect of combining INCAGN01876 and nivolumab on T-cell proliferation in peripheral blood.........21

Figure S5. Effect of combining INCAGN01876 and nivolumab on Treg frequency and proliferation in peripheral blood..22

Figure S6. INCAGN01876 serum preinfusion trough concentration (mean ± SE) from cycles 1 to 12 by dose level. 23

Figure S7. INCAGN01876 serum concentration-time profiles (mean ± SE) by dose level: semilog scale. 24

Supplementary Methods

Patients

Eligible tumor types for phase 1 were advanced or metastatic cervical cancer, endometrial cancer, gastric cancer (including stomach, oesophageal, and gastroesophageal junction), hepatocellular carcinoma, melanoma (excluding uveal melanoma), Merkel cell carcinoma, mesothelioma, microsatellite instability-high colorectal cancer, non-small cell lung cancer, ovarian cancer, squamous cell carcinoma of the head and neck, small cell lung cancer, renal cell carcinoma, triple-negative breast cancer, and urothelial carcinoma or alternative tumor types with medical monitor approval. For patients with cervical cancer, ≤2 prior therapy regimens for advanced or metastatic disease were allowed (not including neoadjuvant and/or adjuvant therapy). Patients with gastric cancer, gastroesophageal junction adenocarcinoma (GEJ), or squamous cell carcinoma of the head and neck (SCCHN) were allowed only 1 prior chemotherapy regimen for advanced or metastatic disease (not including neoadjuvant and/or adjuvant therapy); for patients with SCCHN, this was required to be a platinum-containing chemotherapy regimen. Patients with SCCHN who were enrolled in Simon stage 2 had progressive disease per Response Evaluation Criteria in Solid Tumours (RECIST) v1.1 after ≤6 months of treatment with the platinum-based therapy, which was the last therapy before enrollment. For patients with cervical cancer, gastric cancer, or SCCHN, adjuvant regimens given within 6 months of screening were counted as first-line therapy, and prior treatment with an immune therapy was not allowed. Patients with programmed cell death protein-1 (PD-1)/programmed death ligand 1 (PD-L1) relapsed melanoma had received the anti-PD-1/-PD-L1 therapy as the last therapy prior to enrollment.

Additional exclusion criteria

Patients with laboratory or medical history parameters outside predefined ranges were excluded from the study. Other exclusion criteria included prior treatment with a tumor necrosis factor super family (TNFSF) agonist for any indication, or any anticancer therapy within protocol-defined intervals; not having recovered to grade ≤1 from toxic effects of prior therapy with protocol-defined exceptions; presence of an active autoimmune disease that required previous systemic treatment with protocol-defined exceptions; presence of known active CNS metastases and/or carcinomatous meningitis or active noninfectious pneumonitis or history of interstitial lung disease, hepatitis B or C infection or risk of reactivation, or history of human immunodeficiency virus or history or presence of an abnormal ECG that, in the investigator's opinion, was clinically meaningful. Patients were not allowed to receive any concomitant radiation therapy or live vaccines within 30 days before the first dose of the study treatment and while participating in the study.

Ethics

The protocol (including amendments) was approved by a qualified Institutional Review Board/Independent Ethics Committee prior to patient enrollment at each study site: USA: Western Institutional Review Board, Puyallup, WA (5 sites); The University of Texas MD Anderson Cancer, Center Institutional Review Board, Houston, TX; Memorial Sloan Kettering Cancer Center Institutional Review Board/Privacy Board, New York, NY; IntegReview Ethical

Review Board, Austin, TX (2 sites); Mary Crowley Medical Research Center Institutional Review Board. Dallas, TX; Wayne State University Institutional Review Board Detroit, MI; Providence St. Joseph Health Institutional Review Board, Portland, OR; Washington University School of Medicine Human Research Protection Office (HRPO), St. Louis, MO; Spain: Comité de Ética de la Investigación con Medicamentos - Hospital Universitario, Germans Trias i Pujol, Barcelona, Spain (9 sites); Belgium: Comité d'éthique – Institut Jules Bordet, Anderlecht (7 sites); Australia: Greenslopes Research and Ethics Committee, Greenslopes Private Hospital, Greenslopes QLD; Bellberry Human Research Ethics Committee Eastwood SA (2 sites).

Dose-escalation rules

Dose-escalation decisions and determination of maximum tolerated dose (MTD) were based on treatment-emergent adverse events (TEAEs) observed during the dose-limiting toxicity (DLT) evaluation period (28 days from either cycle 1 day 1 for concurrent dosing or cycle 3 day 1 for sequential dosing). DLTs were defined as the occurrence of any toxicity event, except for events clearly associated with the underlying disease, disease progression, concomitant medication, or comorbidity.

Dose escalation in phase 1 followed a 3+3+3 design algorithm. The first 3 evaluable patients enrolled in the lowest dose cohort were observed for the specified 28-day DLT observation period before patients were enrolled into the next-highest-dose cohort. If no patients experienced DLTs, then enrollment for the next-highest-dose cohort would begin, and a cohort of 3 evaluable patients would be treated at this dose level. If 1 patient in the first cohort of 3 experienced a DLT, the cohort would be expanded to 6 evaluable patients. If 1 of 6 evaluable patients experienced a DLT, a new cohort of 3 evaluable patients would be treated at the next-highest-dose level. If 2 of 6 evaluable patients experienced a DLT, the cohort would be expanded to 9 evaluable patients. If 2 of 9 evaluable patients experienced a DLT, a new cohort of 3 evaluable patients would be treated at the next-highest-dose level. Based on the 3+3+3 algorithm, a minimum of 3 and a maximum of 9 evaluable patients would be enrolled at each dose level within the treatment groups. The pharmacologically active dose was defined as a dose that provided maximal biochemical effect, or an increase in biomarkers of INCAGN0186-induced immune activity, when given in combination therapy.

Simon 2-stage design

In phase 2, a Simon 2-stage design was used. Approximately 16-21 patients per cohort were planned to be enrolled to determine whether the target response rates were likely (**Table, below**). Target response rates were based on historical response rates. An initial number of patients were enrolled in Simon stage 1. The Simon 2-stage design has a stopping rule to allow termination of enrollment to tumor-specific cohorts at the end of stage 1 if insufficient responses were observed in stage 1, as this would indicate that the true response rate is not likely to be ≥ the target rate. In cohorts where ≥ the target responses (ie, sufficient responses) were observed at the end of stage 1, a specified number of additional evaluable patients would be enrolled into stage 2 to allow the true response rate to be estimated. Probabilities of response were calculated based on a 1-sided type 1 error of 0.05 and a power of 85% to determine whether the target response rates were likely to be achieved.

Table. Planned Simon 2-stage designs for phase 2.

Indication	Combination	r_1	n_1	r	n_2	n	p_0	p_1
SCCHN	GITR + Nivo	3	16	10	30	46	15%	35%
Gastric	GITR + Nivo	3	16	10	30	46	15%	35%
Cervical	GITR + Nivo	4	17	14	34	51	20%	40%
Relapsed melanoma	GITR + Nivo	2	20	8	30	50	10%	25%
Relapsed melanoma	GITR + Ipi	2	20	8	30	50	10%	25%
SCCHN	GITR + Nivo + Ipi	5	18	17	32	50	25%	45%
Gastric	GITR + Nivo + Ipi	5	18	17	32	50	25%	45%
Cervical	GITR + Nivo + Ipi	7	21	19	27	48	30%	50%

r_1: If r_1 or fewer responses are observed during stage 1, the study cohort is stopped early for futility.
n_1: Number of participants initially enrolled in stage 1.
r: If r or fewer responses are observed by the end of stage 2, then no further investigation of the drug combination is warranted in the selected tumor type.
n_2: Number of participants enrolled in stage 2.
n: Total number of participants.
p_0: Insufficient response rate.
p_1: Target response rate.

Integrated Bayesian analysis

An integrated Bayesian analysis was also used for the cervical cancer, gastric cancer, and SCCHN cohorts, where pooling of patient data was performed within dosing strategies and tumor types to determine INCAGN01876 activity within any of the given dosing strategies, given tumor types, or given dosing strategy/tumor type combinations.

Biopsy cohorts

For the biopsy cohorts, if a predictive biomarker with 33% prevalence is associated with response, enrollment of 5 patients provides an 86.5% probability of enrolling ≥1 patient with the predictive biomarker in the cohort in question.

Tumor imaging

The same imaging technique was used for patients throughout the study. Initial tumor imaging was performed ≤28 days before the first dose of study treatment to confirm measurable disease per RECIST v1.1. Imaging assessments were performed ≤8 weeks after the first dose of INCAGN01876, then every 8 weeks for 12 months, then every 12 weeks until documented disease progression, start of new anticancer treatment, death, withdrawal of consent, or end of study. Where radiological imaging showed progressive disease, this was confirmed by repeat tumor assessment ≥4 and ≤6 weeks later. Patients considered clinically stable could continue treatment until confirmation. Clinically stable was defined as absence of clinically significant signs and symptoms (including worsening laboratory values) indicating progressive disease, and no decline in Eastern Cooperative Oncology Group (ECOG) performance status, and absence of

rapid progression of disease, and absence of progressive tumor at a critical anatomical site (eg, cord compression) that would require urgent alternative medical intervention. For patients who continued treatment, the date of disease progression was considered the date of the scan confirming disease progression.

Pharmacokinetic (PK) and antidrug antibody (ADA) analyses

Serum samples were collected for PK and ADA analysis for all treatment groups with concurrent dosing (ie, treatment group [TG] A, C, D, C2, and F) preinfusion at day 1 of cycles 1, 2, 4, 6, 8, and 12. An untimed sample was taken at the first safety follow-up visit; samples were also collected for PK analysis postinfusion on day 1 of cycles 1 and 6 (within 10 minutes of INCAGN01876 infusion, before infusion of other agents) and preinfusion on day 1 of cycle 7, and untimed PK samples were also taken on day 8 of cycles 1 and 6. Samples were collected as above for TGB preinfusion on day 1 of cycles 1, 2, and 4 and immediately after infusion on day 1 of cycle 1. Untimed samples were collected on day 8 of cycle 1 and at the first safety follow-up visit.

Plasma protein biomarker analysis and whole-blood T-cell population profiling

Samples were collected for whole-blood lymphocyte assessment preinfusion on day 1 of cycles 1-4, 8, and 12. Samples were also collected on day 8 of cycle 1 and at end of treatment (any time). Samples were collected for plasma correlative analysis preinfusion on day 1 of cycles 1 and 2, at any time on day 8 of cycle 1, and postinfusion ≤10 minutes after INCAGN01876 infusion and before infusion of other agents on day 1 of cycle 1, and ≤60 minutes after INCAGN01876 infusion on day 1 of cycle 2. Samples were collected for whole-blood correlative DNA and RNA analysis (including gene expression analysis and circulating T-cell repertoire clonality and diversity analysis) at screening (any time) and preinfusion at day 1 of cycle 3.

Immunohistochemistry analysis of glucocorticoid-induced tumor necrosis factor receptor (GITR) and PD-L1 in pretreatment tumor biopsy samples

PD-L1 analysis of tumor biopsy samples used mouse clone 22C3, per in vitro diagnostic instructions provided by Agilent Dako (Santa Clara, CA, USA). The GITR immunohistochemistry assay used rabbit clone D5V7. Both assays used DAB chromogen, with Dako Autostainer Link 48 (Agilent Dako). GITR status was determined by retrospective batch testing by a central laboratory. GITR tumor proportion score (TPS) is defined as 100 × (total number of GITR-positive tumor cells/total number of viable tumor cells); GITR immune score was defined as 100 × (total number of GITR-positive immune cells/total number of viable immune cells).

Statistical analyses

PK parameters were summarized based on patient samples collected immediately postinfusion during cycles 1 and 6. Dose proportionality of INCAGN01876 was assessed using a power function model that regresses log-transformed PK exposure versus log-transformed dose. The slope parameter on ln (dose), ie, the exponent on dose in the linear scale, and the 90% CIs were estimated. The Kruskal-Wallis test was used to examine the statistical significance of the median treatment difference in time to maximum serum concentration (t_{max}). Steady state was

determined by analysis of preinfusion trough concentration by visit using The Helmert contrast.[50] The pharmacodynamic (PD)-evaluable population included patients who received ≥1 dose of INCAGN01876 and had ≥1 postdose PD blood sample collected and analyzed. Standard noncompartmental PK methods were used to analyze INCAGN01876 PK data using Phoenix WinNonlin v8.3.4 (Certara USA Inc, Princeton, NJ, USA). Maximum observed serum concentration (C_{max}), minimum observed serum concentration, and t_{max} values were taken directly from observed serum concentration data. Significant differences were defined as $P \leq .05$.

The terminal elimination rate constant (kz) was estimated using a log-linear regression of the concentration data in the terminal disposition phase. The apparent terminal half-life ($t_{1/2}$) was estimated as ln(2)/k. Area under the serum concentration-time curves (AUCs) were estimated using the linear trapezoidal rules for increasing concentrations and the log trapezoidal rules for decreasing concentrations. AUC extrapolated to infinity (AUC_{inf}) was calculated as AUC_{last} + C_{last}/k_z, and the accumulation ratio was computed as the ratio of AUC over the dosing interval (ie, AUC from time 0 to the last measurable sample [AUC_{last}]) on cycle 1 day 1 compared with cycle 6 day 1. Clearance (CL) was calculated as dose/AUC_{inf} or dose/AUC from time 0 to end of the dose interval (AUC_{tau}) at cycles 1 and 6, respectively, and apparent volume of distribution during the terminal phase (V_z) was calculated as $V_z = CL/k_z$.

Supplementary Results

Patients

Among patients in TGF with SCCHN, the most common sites of primary tumors at initial diagnosis were oral cavity (n=14, 30.4%) and oropharynx (n=12, 26.1%), and tumors were most commonly Stage 4A (39.1%). Most patients (n=30, 65.2%) had 1 line of prior cancer therapy; the most common prior anticancer systemic therapy was platinum-based chemotherapy (n=45, 97.8%). A positive GITR TPS was reported for 39.1% of patients, and human papillomavirus (HPV) status was positive/mutant for 13%; however, values were missing for 34.8% and 60.9% of patients for these respective tests. All patients in the study discontinued treatment, primarily due to disease progression: phase 1, n=41 (80.4%); phase 2, TGC2, n=6 (75.0%); TGF, n=56 (65.1%).

Exposure

For patients with SCCHN in TGF, median (range) number of INCAGN01876 and nivolumab infusions was 9 (1-52) and 9 (1-52), respectively. Mean (SD) duration of treatment with INCAGN01876 and nivolumab was 223.2 (249.0) days and 223.5 (249.3) days, respectively. Mean (SD) average dose of INCAGN01876 and nivolumab was 4.6 (0.9) mg/kg and 3.7 (0.8) mg/kg, respectively.

Safety

In phase 1, TEAEs were reported in all patients in TGA, B, and D, and 93.3% of patients in TGC. The most common TEAEs in phase 1 were pruritus (n=12, 23.5%), anemia (n=11, 21.6%), rash, and nausea (n=10, 19.6% each). (**Table S5**). . In phase 2, TEAEs were reported in all patients in TGC2, most commonly fatigue (n=6, 75.0%), and in 98.8% of patients in TGF, most commonly fatigue (n=26, 30.2%) (**Table S5**). DLTs were reported for 1 patient in phase 1 (2.0%, TGD; nonserious grade 3 pruritus and nonserious grade 3 rash) and 3 patients in phase 2 (1 patient [12.5%] in TGC2, nonserious grade 2 penile pain and nonserious grade 2/3 pruritus; and 2 patients [2.3%] in TGF, nonserious grade 2/3 rash and serious grade 3 bronchial obstruction, n=1 patient each). No dose-dependent toxicities were observed, and MTD was not reached. TEAEs leading to dose interruption (defined as <4 weeks) or delay or treatment discontinuation are shown in **Table 3**. Investigator-assessed INCAGN01876-related TEAEs (TRAEs) were reported in 29 patients (56.9%) in phase 1, most commonly pruritus (n=9, 17.6%) and rash (n=8, 15.7%), and in 62 patients (66.0%) in phase 2, most commonly fatigue and pruritus (n=15, 16.0% each). In phase 2, grade ≥3 TRAEs were reported for 1 patient in TGC2 and 16 patients in TGF; all events n=1 per TG (**Table 3**). The most frequently reported any-grade and grade ≥3 TEAEs and TRAEs across all TGs are detailed in **Tables S5** and **S6**. The most common immune-related TEAEs (irAEs) were pruritus (phase 1, n=10, 19.6%; phase 2, n=7, 13.0%) and rash (phase 1, n=8, 15.7%; phase 2, n=4, 4.3%; **Table S7**).

PK analyses

PK analysis was performed across all TGs on 754 samples from 143 of 145 patients dosed with INCAGN01876. Data were available for 141 patients. Based on trough serum concentrations,

steady state was reached at approximately cycle 6 (**Figure S6**). Mean (± SE) serum concentration-time profiles by dose regimen on cycle 1 day 1 and cycle 6 day 1 (steady state) for all individuals are shown in **Figure S7**. PK parameters at steady state (n=55), by dosing level, are detailed in **Table S8**. Geometric mean (SD) C_{max} was 145 mg/L at steady state. Median (range) t_{max} across all dose levels was 0.6 hours (0.4-2.4) after the first dose and 0.6 hours (0.5-1.3) at steady state. At steady state, geometric mean $t_{½}$ was 214 hours, or approximately 9 days, across dose regimens. Geometric mean accumulation ratios were 1.43 and 1.78 for C_{max} and AUC, respectively. Based on steady state data obtained during cycle 6, INCAGN01876 exposure was dose proportional across all dose levels tested for both C_{max} (slope = 1.136 [95% CI, 0.919-1.352]) and AUC_{tau} (1.148 [95% CI, 0.905-1.392]). For the recommended phase 2 dose of 300 mg every 2 weeks, geometric mean (SD) C_{max} was 145 mg/L, the median (range) t_{max} was 0.6 hours (0.5-1.3), geometric mean of AUC_{tau} was 28,500 h•mg/L, and geometric mean $t_{½}$ was 212 hours.

ADA analyses

All ADA-positive patients were treatment emergent (ie, ADA negative at baseline but tested positive postbaseline), and no treatment-boosted ADA incidences were observed in this study. No patients receiving INCAGN01876 10 mg/kg (n=4) tested positive for ADAs.

Supplemental Tables

Table S1. Definition of DLTs.[a]

**Nonhematologic toxicity**
• Any nonhematological toxicity at grade ≥3, excluding the following:<br>− Transient (≤72 hours) abnormal laboratory values in the absence of associated clinically significant signs or symptoms.<br>− Nausea, vomiting, and diarrhea that are adequately controlled with supportive care within a duration of 48 hours.<br>− Changes in cholesterol and triglycerides.<br>− Any event clearly associated with the underlying disease, or disease progression, or a concomitant medication, or a comorbidity.<br>− Asymptomatic changes in amylase and lipase.<br>− Singular or nonfasting increases in blood glucose (blood glucose excursions are considered toxicities where fasting blood glucose is elevated on 2 separate occasions).<br>− Grade 3 rash in the absence of desquamation, with no mucosal involvement, that does not require systemic steroids and that resolves to grade 1 within 14 days.
**Hematologic toxicity**
• Grade 3 thrombocytopenia with clinically significant bleeding, ie, bleeding that requires hospitalization, transfusion of blood products, or other urgent medical intervention.<br>• Grade 4 thrombocytopenia.<br>• Grade ≥3 febrile neutropenia (ie, absolute neutrophil count <1.0 × 10^9/L and fever >101 °F/38.3 °C).<br>• Grade 4 neutropenia that does not recover to grade ≤2 within 3 days following interruption of study drug.<br>• Grade 4 anemia that is not due to the underlying disease or some other concomitant disorder.
**Immune-related toxicity**
• Grade ≥2 ocular irAEs[b] will be considered a DLT.<br>• Grade 3 irAEs[a] that do not improve to baseline or at least grade 1 within <5 days with appropriate care or with corticosteroid therapy will be considered a DLT.<br>• Any grade 4 irAEs[a] will be considered a DLT, regardless of duration.
**General**
• An inability to receive the planned number of doses of study drug within the 28-day DLT period due to toxicity will be considered a DLT, regardless of grade.
**Maximum tolerated dose**
• In phase 1 of the study, MTD will be defined as 1 dose level lower than that at which at least one-third of patients in a particular cohort have DLTs.<br>• In phase 2 of the study, toxicities will continue to be monitored. If the cumulative incidence of grade ≥3 INCAGN01876-related AEs occurs in >40% of patients in a particular treatment group after 6 patients have been enrolled, then further enrollment in that treatment group will be stopped until the sponsor, investigators, and regulatory authorities (if applicable) determine the appropriate course of action. If a treatment group is discontinued due to toxicity, it may be restarted at a previously tested lower dose level and/or alternate dosing schedule. All AEs may be considered for DLT determination purposes regardless of the time of occurrence on study.

Error! Reference source not found.28-day DLT observation period.
[b]irAEs are a diagnosis of exclusion, after alternative etiologies have been ruled out.
Abbreviations: AE, adverse event; DLT, dose-limiting toxicity; irAE, immune-related adverse event; MTD, maximum tolerated dose.

Table S2. Summary of exposure.

INCAGN01876 exposure	Phase 1				Phase 2	
	**Treatment group A (n=17)**	**Treatment group B (n=13)**	**Treatment group C (n=15)**	**Treatment group D (n=6)**	**Treatment group F (n=86)**	**Treatment group C2 (n=8)**
Treatment infusions, n, median (range)	4.0 (1-53)	2.0 (2-2)	4.0 (2-27)	4.0 (2-6)	8.0 (1-52)	7.0 (2-16)
Duration of treatment, days, mean (SD)	167.9 (251.7)	15.1 (0.6)	113.5 (118.8)	41.7 (18.2)	168.4 (206.2)	97.3 (60.5)
Average dose, mg/kg, mean (SD)	4.8 (3.3)	2.6 (1.6)	2.9 (1.4)	1.0 (0.0)	4.5 (1.1)	3.8 (0.5)
**INCAGN01876 exposure**	**Phase 2**					
	**Gastric cancer (n=16)**	**SCCHN (n=46)**	**Cervical cancer (n=18)**	**PD-(L)1 relapsed melanoma (n=4)**	**Biopsy (n=2)**	
Treatment infusions, median (range)	5.0 (1-13)	9.0 (1-52)	5.5 (1-40)	7.5 (2-18)	4.5 (1-8)	
Duration of treatment, days, mean (SD)	73.8 (53.7)	223.2 (249.3)	135.2 (152.8)	124.5 (119.7)	53.5 (74.3)	
Average dose, mg/kg, mean (SD)	4.2 (0.9)	4.6 (0.9)	4.8 (1.5)	3.2 (0.9)	3.9 (1.9)	
**Nivolumab exposure**						
Treatment infusions, n, median (range)	5.0 (1-13)	9.0 (1-52)	5.5 (1-40)	7.5 (2-18)	4.5 (1-8)	
Duration of treatment, days, mean (SD)	73.8 (3.6)	223.5 (249.3)	135.2 (152.8)	124.5 (119.7)	53.5 (74.3)	
Average dose, mg/kg, mean (SD)	3.4 (0.8)	3.7 (0.8)	3.9 (1.2)	2.6 (0.7)	3.1 (1.5)	

Abbreviations: SCCHN, squamous cell carcinoma of the head and neck; SD, standard deviation.

Table S3. Summary of efficacy endpoints. Data represent unconfirmed tumor responses (phase 1) and confirmed tumor responses (phase 2) according to RECIST v1.1.

Variable	Phase 1				Phase 2	
	Treatment group A (n=17)	Treatment group B (n=13)	Treatment group C (n=15)	Treatment group D (n=6)	Treatment group F (SCCHN only) (n=46)	Treatment group C2 (n=8)
**DCR, n (%)[a,b]**	6 (35.3)	3 (23.1)	5 (33.3)	0	25 (54.3)	3 (37.5)
Median duration of disease control, days (95% CI)[a,c]	624.0 (76.0-NE)	167.0 (125.0-NE)	276.0 (110.0-NE)	NE (NE-NE)	253.0 (156.0-NE)	218.0 (106.0-NE)
Median DOR, days (95% CI)[a,d]	NE (573.0-NE)	876.0 (NE-NE)	281.0 (NE-NE)	NE (NE-NE)	NE (118.0-NE)	NE (NE-NE)
**PFS[e,f]**						
Median PFS, days (95% CI)	55.0 (48.0-76.0)	57.0 (38.0-125.0)	56.0 (52.0-225.0)	54.5 (21.0-NE)	115.0 (59.0-174.0)	57.0 (20.0-218.0)
Observed - death (%)	2 (11.8)	0	0	1 (16.7)	4 (8.7)	1 (12.5)
Observed - disease progression, n (%)	12 (70.6)	12 (92.3)	13 (86.7)	5 (83.3)	30 (65.2)	7 (87.5)
Censored, n (%)	3 (17.6)	1 (7.7)	2 (13.3)	0	12 (26.1)	0
12-month survival rate (95% CI)	19.3 (4.8-41.0)	8.3 (0.5-31.1)	14.3 (2.3-36.6)	0.0 (NE-NE)	25.3 (13.3-39.3)	0.0 (NE-NE)
**OS[e,f]**						
Median OS, days (95% CI)	253.0 (76.0-NE)	419.0 (170.0-519.0)	298.0 (128.0-1076.0)	229.5 (46.0-NE)	491.0 (344.0-NE)	290.5 (20.0-NE)
Deaths, n (%)						
Observed	11 (64.7)	10 (76.9)	11 (73.3)	6 (100.0)	25 (54.3)	6 (75.0)
Censored, n (%)	6 (35.3)	3 (21.1)	4 (26.7)	0	21 (45.7)	2 (25.0)
12-month survival rate (95% CI)	34.0 (11.6-58.2)	50.3 (21.1-73.9)	46.8 (19.6-70.2)	16.7 (0.8-51.7)	57.0 (40.7-70.4)	50.0 (15.2-77.5)
24-month survival rate (95% CI)	25.5 (6.7-50.2)	10.1 (0.6-35.7)	31.2 (9.7-55.9)	16.7 (0.8-51.7)	40.5 (24.8-55.6)	37.5 (8.7-67.4)

[a]Response-evaluable population.
[b]Disease control included patients who had a BOR of SD (≥49 days), confirmed PR, or CR.
[c]Duration of disease control as measured from the start of treatment until disease progression or death from any cause, if occurring sooner than progression, was determined by investigator assessment of radiographic disease per RECIST v1.1.
[d]Duration of response was the time from the first overall response contributing to an objective response (CR or PR) to the earlier of the patient's death from any cause or first assessment of PD.
[e]Full analysis set.
[f]Median survival time was estimated using the Kaplan-Meier method.
Abbreviations: BOR, best overall response; CR, complete response; DCR, disease control rate; DOR, duration of response; NE, not evaluable; OS, overall survival; PD, progressive disease; PFS, progression-free survival; PR, partial response; RECIST, Response Evaluation Criteria in Solid Tumours; SCCHN, squamous cell carcinoma of the head and neck; SD, stable disease.

Table S4. Summary of efficacy endpoints in the phase 2 treatment group F cohorts other than SCCHN. Data represent unconfirmed tumor responses (phase 2) according to RECIST v1.1.

Variable	Treatment group F			
	**Gastric cancer (n=16)**	**Cervical cancer (n=18)**	**PD-1/PD-L1 relapsed melanoma (n=4)**	**Biopsy (n=2)**
DCR, n (%)[a,b]	9 (56.3)	10 (55.6)	2 (50.0)	1 (50.0)
Median duration of disease control, days (95% CI)[a,c]	109.0 (63.0-NE)	168.0 (71.0-NE)	206.5 (118.0-NE)	109.0 (NE-NE)
Median DOR, days (95% CI)[a,d]	NE (NE-NE)	116.5 (50.0-NE)	NE (NE-NE)	NE (NE-NE)
Median PFS, days (95% CI)[e,f]	75.0 (45.0-120.0)	102.0 (51.0-225.0)	87.0 (52.0-NE)	64.5 (20.0-NE)
Median OS, days (95% CI)[e,f]	179.0 (75.0-404.0)	492.0 (92.0-733.0)	485.0 (52.0-NE)	264.0 (20.0-NE)

[a]Response-evaluable population.
[b]Disease control included patients who had a BOR of SD (≥49 days), confirmed PR, or CR. BOR was defined as the best response recorded before and including the first event of PD in the order of CR, PR, SD (≥49 days), PD, and NE.
[c]Duration of disease control as measured from the start of treatment until disease progression or death from any cause, if occurring sooner than progression, was determined by investigator assessment of radiographic disease per RECIST v1.1.
[d]Duration of response was the time from the first overall response contributing to an objective response (CR or PR) to the earlier of the patient's death from any cause or first assessment of PD.
[e]Full analysis set.
[f]Median survival time was estimated using the Kaplan-Meier method.
Abbreviations: BOR, best overall response; CR, complete response; DCR, disease control rate; DOR, duration of response; NE, not evaluable; OS, overall survival; PD, progressive disease; PD-1, programmed cell death protein 1; PD-L1, programmed cell death ligand 1; PFS, progression-free survival; PR, partial response; RECIST, Response Evaluation Criteria in Solid Tumours; SCCHN, squamous cell carcinoma of the head and neck; SD, stable disease.

Table S5. TEAEs.[a]

MedDRA Preferred Term, n (%)	Any-grade TEAEs
**Treatment group A (n=17)**	
Abdominal distention	3 (17.6)
Anemia	3 (17.6)
Arthralgia	3 (17.6)
AST increased	3 (17.6)
Cough	3 (17.6)
Dyspnea	3 (17.6)
Fatigue	3 (17.6)
Headache	3 (17.6)
Pruritus	3 (17.6)
**Treatment group B (n=13)**	
Rash	5 (38.5)
Arthralgia	3 (23.1)
Back pain	3 (23.1)
Fatigue	3 (23.1)
Pruritus	3 (23.1)
**Treatment group C (n=15)**	
Nausea	8 (53.3)
Vomiting	6 (40.0)
Pruritus	5 (33.3)
Constipation	4 (26.7)
Decreased appetite	4 (26.7)
Anemia	3 (20.0)
Back pain	3 (20.0)
Cough	3 (20.0)
Diarrhea	3 (20.0)
Dyspnea	3 (20.0)
Nasopharyngitis	3 (20.0)
Pain in extremity	3 (20.0)
Rash	3 (20.0)
Upper respiratory tract infection	3 (20.0)
**Treatment group D (n=6)**	
Anemia	3 (50.0)
Decreased appetite	3 (50.0)
**Treatment group C2 (n=8)**	
Fatigue	6 (75.0)
Cough	3 (37.5)
Nausea	3 (37.5)
Pruritus	3 (37.5)
**Treatment group F (n=86)**	
Fatigue	26 (30.2)
Diarrhea	22 (25.6)
Nausea	22 (25.6)
Pruritus	21 (24.4)
Cough	16 (18.6)
Anemia	15 (17.4)
Vomiting	14 (16.3)
Arthralgia	13 (15.1)
Constipation	13 (15.1)

MedDRA Preferred Term, n (%)	Any-grade TEAEs
Dyspnea	13 (15.1)
Abdominal pain	12 (14.0)
Decreased appetite	12 (14.0)
Edema peripheral	11 (12.8)
Asthenia	10 (11.6)
Pyrexia	10 (11.6)
Rash	10 (11.6)

[a]Occurring in ≥3 patients in treatment groups A, B C, and D and treatment group C2, and in ≥10 patients in treatment group F.

Abbreviations: AST, aspartate aminotransferase; MedDRA, Medical Dictionary for Regulatory Activities; TEAE, treatment-emergent adverse event.

Table S6. Grade ≥3 TEAEs.[a]

MedDRA Preferred Term, n (%)	Grade ≥3 TEAEs
**Treatment group A (n=17)**	
Pneumothorax	2 (11.8)
**Treatment group B (n=13)**	
Anemia	2 (15.4)
**Treatment group C (n=15)**	
Anemia	3 (20.0)
**Treatment group D (n=6)**	None reported in ≥2 patients
**Treatment group C2 (n=8)**	None reported in ≥2 patients
**Treatment group F (n=86)**	
Anemia	6 (7.0)
Abdominal pain	5 (5.8)
Pneumonia	5 (5.8)
Dysphagia	4 (4.7)
Pulmonary embolism	4 (4.7)
Vomiting	3 (3.5)
Hypercalcemia	3 (3.5)
Acute myocardial infarction	2 (2.3)
Asthenia	2 (2.3)
Fatigue	2 (2.3)
General physical health deterioration	2 (2.3)
Urinary tract infection	2 (2.3)
Lipase increased	2 (2.3)
Dyspnea	2 (2.3)
Pneumonitis	2 (2.3)
Hypertension	2 (2.3)

[a]Occurring in ≥2 patients.
Abbreviations: MedDRA, Medical Dictionary for Regulatory Activities; TEAE, treatment-emergent adverse event.

Table S7. irAEs.[a]

MedDRA Preferred Term, n (%)	Any-grade irAEs
**Treatment group A (n=17)**	
Any event	5 (29.4)
Pruritus	2 (11.8)
**Treatment group B (n=13)**	
Any event	5 (38.8)
Rash	5 (38.5)
Pruritus	3 (23.1)
**Treatment group C (n=15)**	
Any event	6 (40%)
Pruritus	4 (26.7)
Rash	2 (13.3)
**Treatment group D (n=6)**	
Any event	2 (33.3)
	None reported in ≥2 patients
**Treatment group C2 (n=8)**	
Any event	3 (27.5)
	None reported in ≥2 patients
**Treatment group F (n=86)**	
Any event	24 (27.9)
Pruritus	6 (7.0)
Rash	4 (4.7)
Hypothyroidism	4 (4.7)
Diarrhea	3 (3.5)
Lymphocyte count decreased	3 (3.5)

[a]Occurring in ≥2 patients.
Abbreviations: irAE, immune-related adverse event; MedDRA, Medical Dictionary for Regulatory Activities.

Table S8. INCAGN01876 PK.[a]

INCAGN01876 Q2W	C_{max}, mg/L, mean ± SD	t_{max}, hours, median (range)	C_{min}, mg/L, mean ± SD	Accumulation index, mean ± SD	AUC_{tau}, h·mg/L, mean ± SD	$t_{½}$, hours, mean ± SD	CL_{ss}, L/h, mean ± SD	V_{ss}, L, mean ± SD
All (n=55)	-	0.6 (0.5-1.3)	-	1.56 ± 0.300 (1.54)	-	226 ± 76.5 (214)	0.0109 ± 0.00337 (0.0104)	3.22 ± 1.16 (2.99)
1 mg/kg (n=2)	34.2, 30.7	0.6, 0.5	9.50, 10.2	1.38, 1.73	6160, 6860	181, 271	0.0120, 0.01	2.97, 4.64
3 mg/kg (n=6)	105 ± 22.8 (103)	0.5 (0.5-0.6)	31.5 ± 10.5 (29.6)	1.45 ± 0.179 (1.44)	20,300 ± 5710 (19,500)	199 ± 48.2 (193)	0.0126 ± 0.00299 (0.0122)	3.25 ± 1.17 (3.05)
5 mg/kg (n=3)	204 ± 15.6 (204)	0.7 (0.5-0.7)	82.2 ± 25.4 (79.6)	1.91 ± 0.200 (1.90)	48,400 ± 7570 (48,000)	313 ± 49.0 (311)	0.00648 ± 0.000428 (0.00647)	2.84 ± 0.378 (2.82)
10 mg/kg (n=1)	386	0.6	104	1.37	70,000	177	0.00846	2.02
300 mg/kg (n=43)	151 ± 47.5 (145)	0.6 (0.5-1.3)	48.7 ± 20.5 (44.6)	1.56 ± 0.312 (1.53)	29,800 ± 9040 (28,500)	225 ± 79.5 (212)	0.0110 ± 0.00340 (0.0105)	3.25 ± 1.21 (3.00)

[a]Parameters were obtained at steady state (cycle 6)are presented as mean ± SD (geometric mean), except t_{max}, which is presented as median (min, max). Individual values are provided if n <3.
Abbreviations: AUC_{tau}, area under the plasma or serum concentration-time curve from time 0 to end of the dose interval; CL_{ss}, steady-state clearance; C_{max}, maximum observed plasma concentration; C_{min}, minimum observed plasma concentration over the dose interval; max, maximum; min, minimum; PK, pharmacokinetics; Q2W, every 2 weeks; $t_{1/2}$, terminal half-life; t_{max}, time to maximum concentration; V_{ss}, steady-state volume of distribution.

Table S9. Summary of immunogenicity results in INCAGN01876.

ADA status, n (%)	Treatment with INCAGN01876 Q2W					All treatments
	1 mg/kg	3 mg/kg	5 mg/kg	10 mg/kg	300 mg/kg	
Evaluable patients[a]	19	16	12	4	94	145
Negative patients[b]	11 (57.9)	12 (75.0)	9 (75.0)	4 (100)	78 (83.0)	114 (78.6)
Treatment-emergent positive patients[c]	8 (42.1)	4 (25.0)	3 (25.0)	0	10 (10.6)	25 (17.2)
Persistent treatment-emergent positive patients[d]	5 (26.3)	4 (25.0)	3 (25.0)	0	4 (4.3)	16 (11.0)

[a]Patients treated with INCAGN01876 with ≥1 ADA sample assayed that was either negative or positive and who did not have a missing ADA screening or confirmatory assay result.
[b]ADA-evaluable patients who did not have positive ADA samples, or had a positive ADA sample at baseline but did not have a postbaseline positive ADA sample, or had a positive baseline ADA sample but did not have a positive postbaseline ADA sample with a titer ≥4-fold of the titer of the positive ADA baseline sample.
[c]ADA-evaluable patients negative at baseline but with ≥1 positive postbaseline ADA sample.
[d]The interval between treatment-emergent ADA samples and the last sample was ≤16 weeks.
Abbreviations: ADA, antidrug antibody; Q2W, every 2 weeks.

Supplemental Figures

Figure S1. Duration of unconfirmed response by RECIST v1.1 (phase 2) among patients in treatment group F with SCCHN. Best overall response and GITR TPS (positive ≥10%, negative <10%) are shown. Abbreviations: CR, complete response; GITR, glucocorticoid-induced tumor necrosis factor receptor; NE, not evaluable; PD, progressive disease; PR, partial response; RECIST, Response Evaluation Criteria in Solid Tumours; SCCHN, squamous cell carcinoma of the head and neck; SD, stable disease; TPS, tumor proportion score.

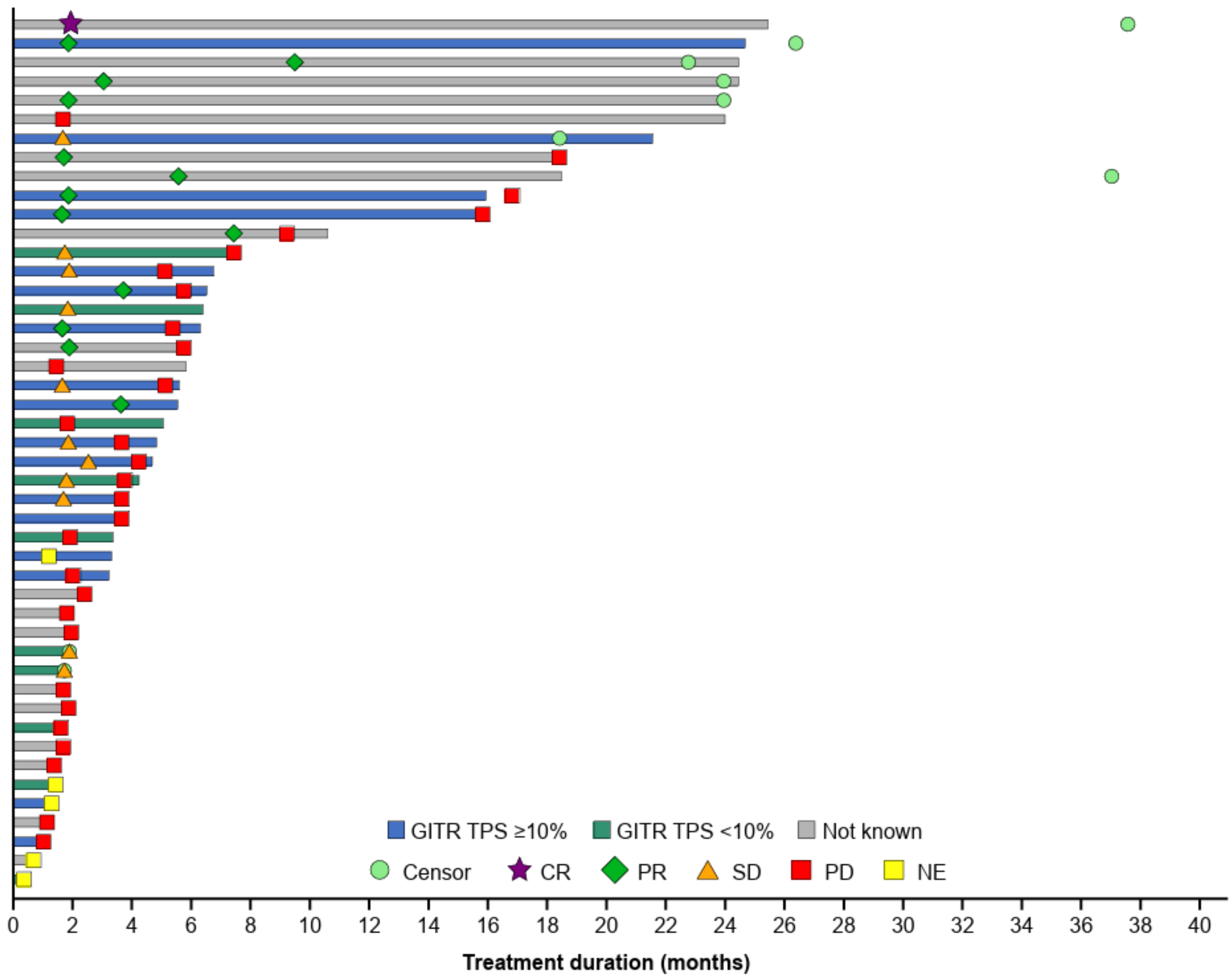

Figure S2. Spider plot of percentage change from baseline in the sum of target lesions over time among patients in treatment group F with SCCHN. Abbreviation: SCCHN, squamous cell carcinoma of the head and neck.

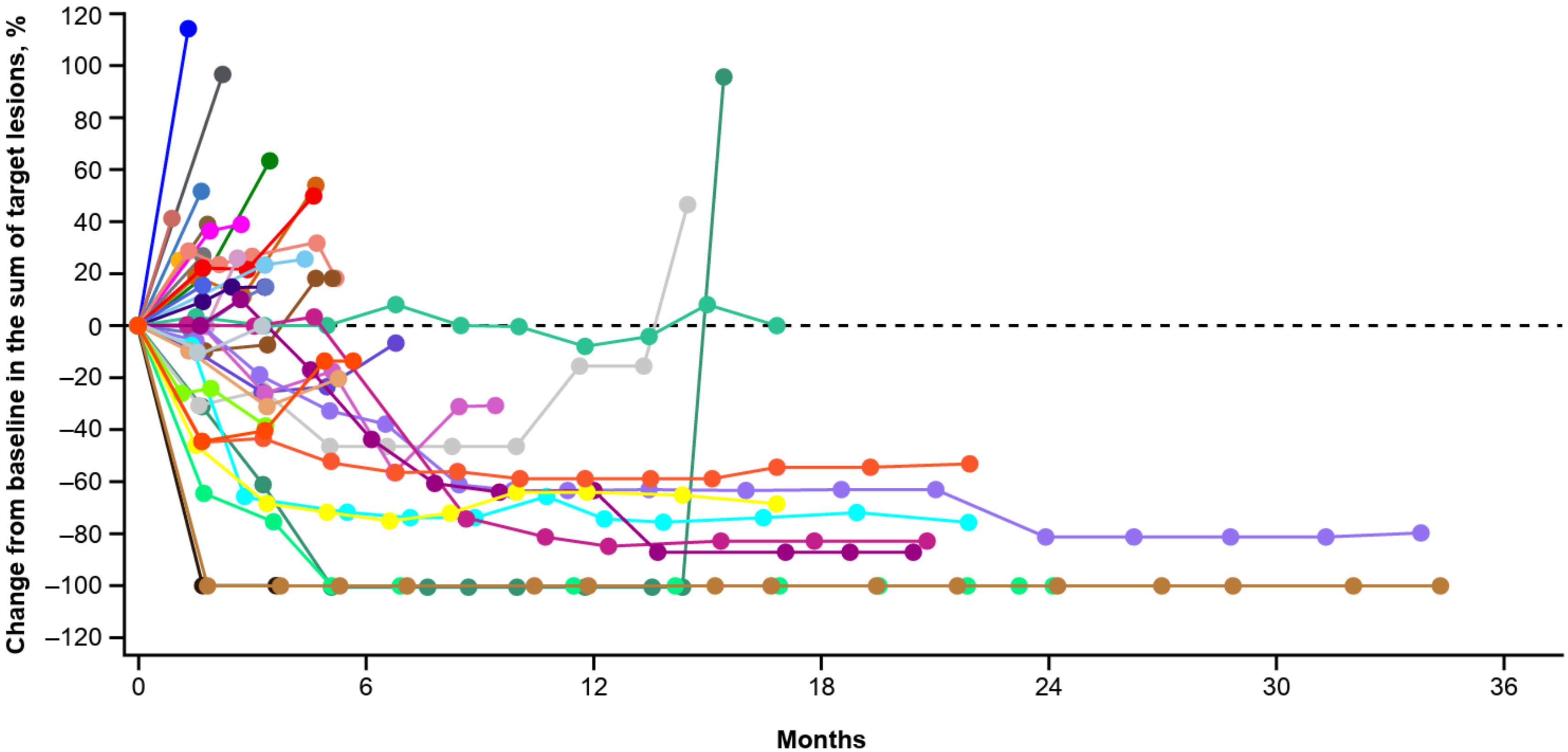

Figure S3. Effect of treatment with INCAGN01876 with nivolumab on cytokine expression among patients in treatment group F with SCCHN or cervical cancer. The analysis includes 15 patients each with evaluable results. Abbreviations: C, cycle; D, day; SCCHN, squamous cell carcinoma of the head and neck.

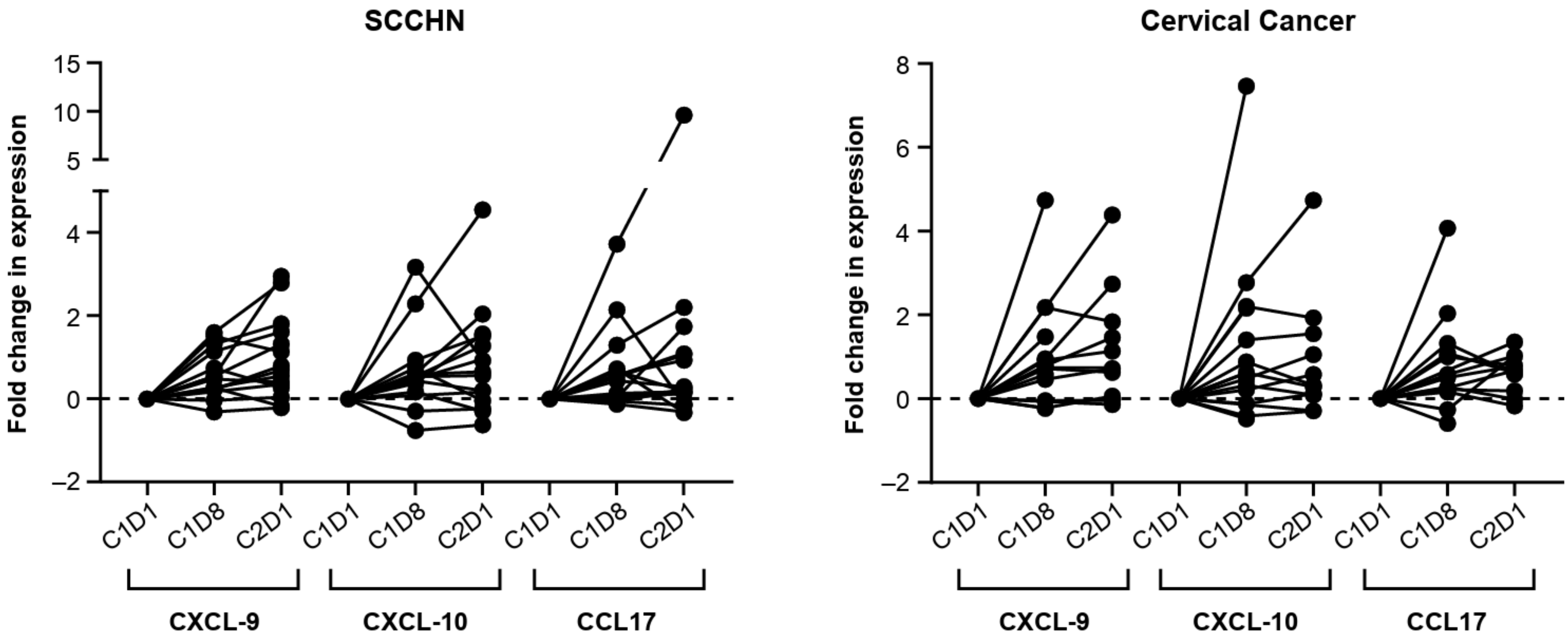

Figure S4. Effect of combining INCAGN01876 and nivolumab on T-cell proliferation in peripheral blood. CD3$^+$ T-cell proliferation in the SCCHN cohort from C1D1 to C8D1 showing median frequencies of Ki67$^+$CD3 cells and standard errors at baseline (C1D1) and later time points. Mean frequency changes and SEM are shown. **P*<.05; ****P*<.001; *****P*<.0001. Abbreviations: C, cycle; D, day; SEM, standard error of the mean.

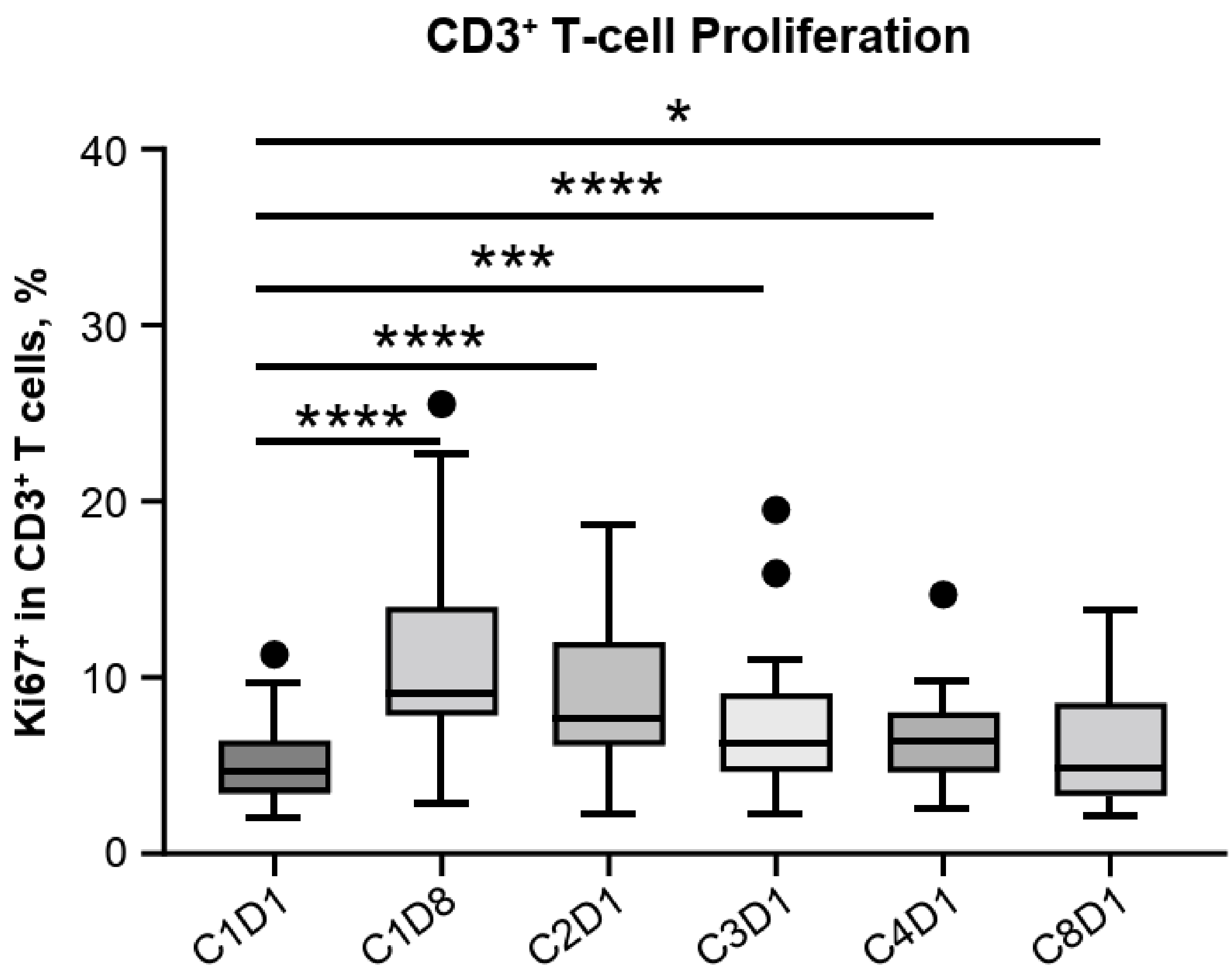

Figure S5. Effect of combining INCAGN01876 and nivolumab on Treg frequency and proliferation in peripheral blood. Treg frequency in the SCCHN cohort, showing (A) median frequencies of Ki67[+]Treg cells and (B) frequency of proliferating Ki67[+]Treg cells from C1D1 (baseline) to C4D1, is shown with standard errors. *$P<.05$; **$P<.01$; ****$P<.0001$. Abbreviations: C, cycle; D, day; ns, not significant; SCCHN, squamous cell carcinoma of the head and neck; Treg, regulatory T cell.

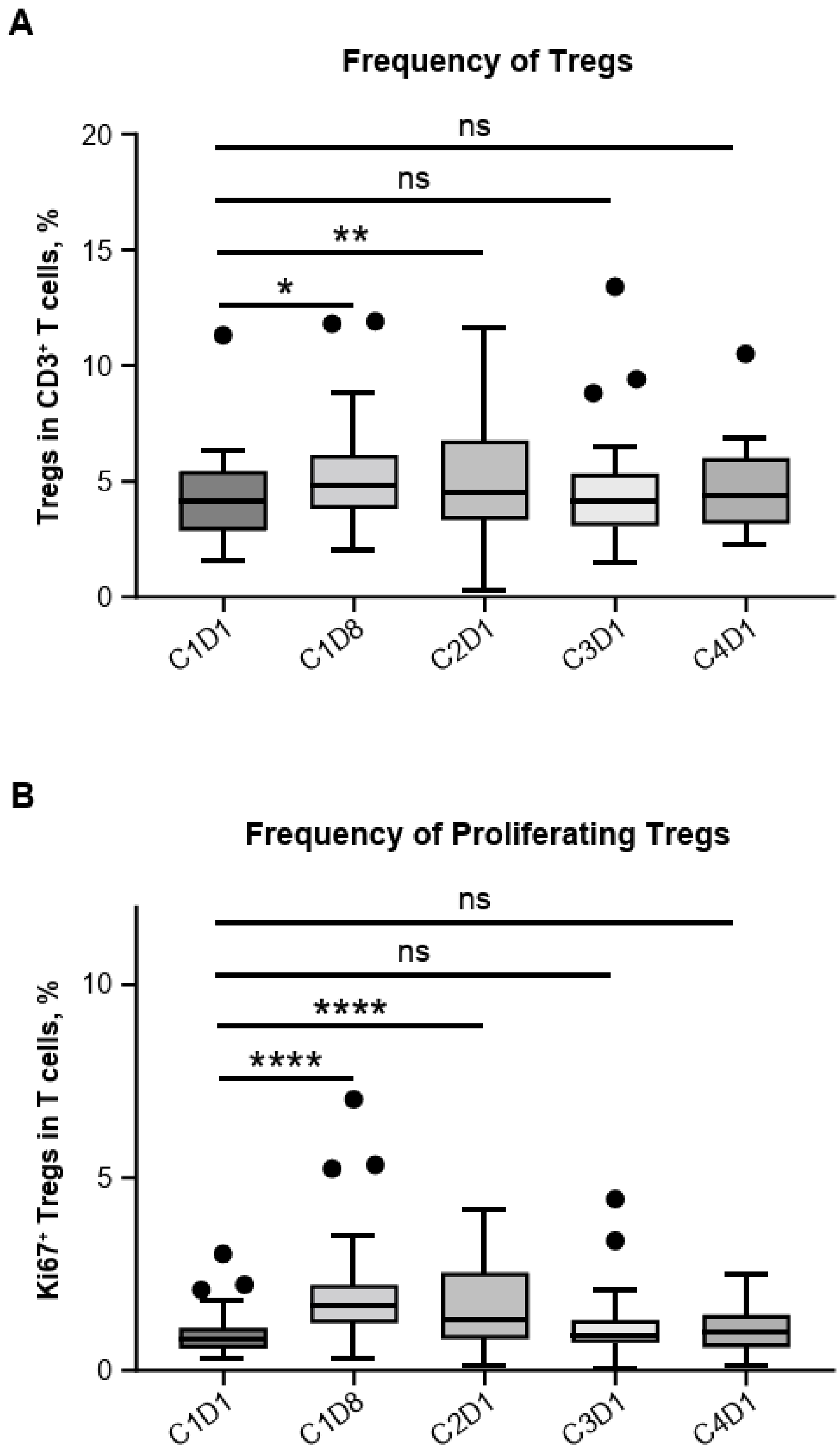

Figure S6. INCAGN01876 serum preinfusion trough concentration (mean ± SE) from cycles 1 to 12 by dose level. Data represent the PK-evaluable population. Abbreviation: PK, pharmacokinetic.

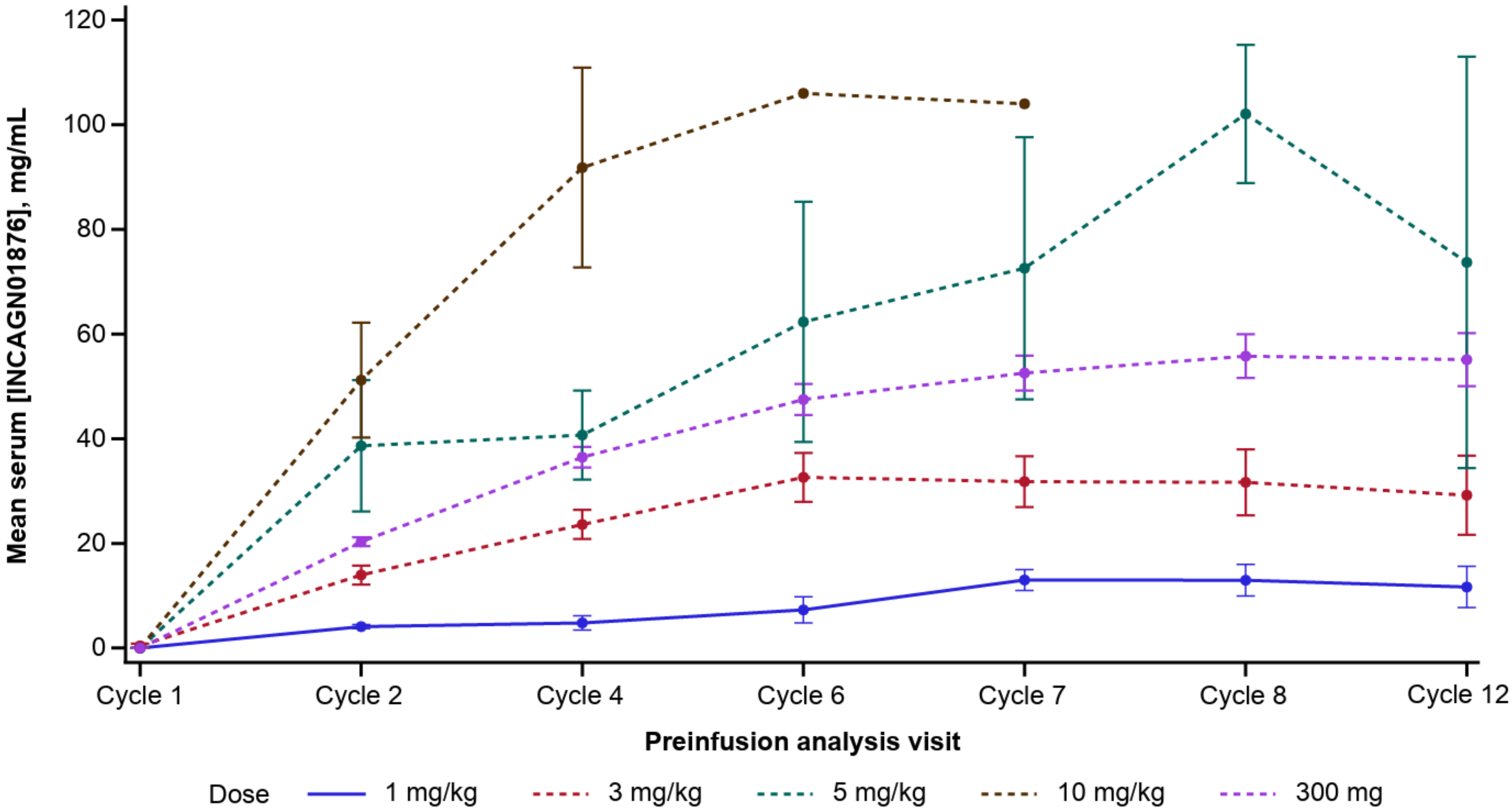

Figure S7. INCAGN01876 serum concentration-time profiles (mean ± SE) by dose level: semilog scale. Data represent the PK-evaluable population. Abbreviation: PK, pharmacokinetic.

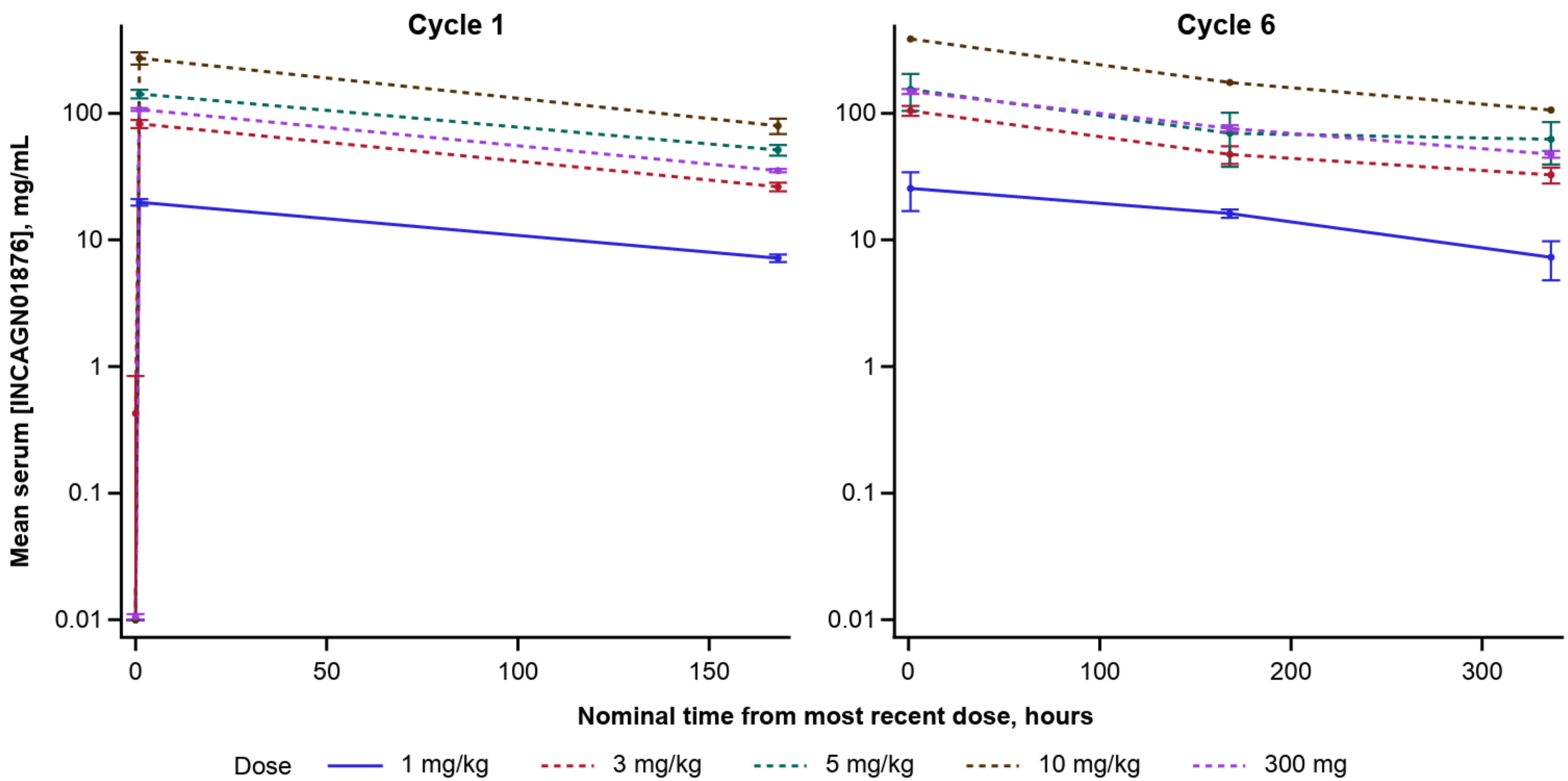

Supplement: Supplementary file 1 [file oyag257_supplementary_data.pdf]
